# Mindfulness training in healthcare professions: A scoping review of systematic reviews

**DOI:** 10.1111/medu.15293

**Published:** 2024-01-17

**Authors:** Nabeela Kajee, Jesus Montero-Marin, Kate E. A. Saunders, Kearnan Myall, Elinor Harriss, Willem Kuyken

**Affiliations:** ^1^ Department of Psychiatry, University of Oxford Warneford Hospital Oxford UK OX3 7JX; ^2^ Teaching, Research & Innovation Unit, Parc Sanitari Sant Joan de Déu Sant Boi de Llobregat Spain; ^3^ Consortium for Biomedical Research in Epidemiology & Public Health (CIBER Epidemiology and Public Health, CIBERESP) Madrid Spain 28029; ^4^ Associate Professor, Department of Psychiatry, University of Oxford Warneford Hospital Oxford UK OX3 7JX; ^5^ Outreach and Enquiry Services Manager, Bodleian Health Care Libraries University of Oxford UK OX3 7JX; ^6^ Professor, Department of Psychiatry, University of Oxford Warneford Hospital Oxford UK OX3 7JX

## Abstract

**Purpose:**

The effectiveness of mindfulness training (MT) on mental health and wellbeing in different groups and contexts is well‐established. However, the effect of MT on different healthcare professionals' (HCPs) mental health and wellbeing needs to be synthesised, along with a focus on outcomes that are specifically relevant to healthcare settings. The aim of this study is to summarise the effect of MT interventions on HCPs' mental health and wellbeing, to explore its effect on communication skills and to identify potential gaps in the literature.

**Methods:**

A scoping review of systematic reviews (SRs) investigating MT interventions in HCPs was conducted. A comprehensive systematic search was conducted from database inception to 22 February 2023 on Ovid MEDLINE, Ovid Embase, Scopus, Cochrane (CENTRAL), EBSCHOhost CINAHL, Ovid PsycINFO, Web of Science (Core Collection), OpenGrey, TRIP Database and Google Scholar. Snowballing of reference lists and hand‐searching were utilised. Risk of bias and quality of included SRs were assessed using the ROBIS and AMSTAR2 tools.

**Results:**

Sixteen SRs were included in this review. We found substantial evidence for MT interventions improving mental health and wellbeing across different HCPs, with the exception of burnout, where evidence is mixed. There is a paucity of SRs evaluating communication skills other than empathy. However, the available evidence is suggestive of improvements in self‐reported empathy. Details of MT fidelity and dosage are largely absent in the SRs, as is study populations from representative EDI samples.

**Conclusions:**

Synthesis of SRs suggests that MT improves mental health and wellbeing in HCPs. The exception is burnout, where results are inconclusive. Insufficient data exists to evaluate effects of MT on the full spectrum of communication skills. Other HCPs than medicine and nursing are inadequately represented. Further research is required that considers the specific target population of HCPs and MT curriculum, and reports on fidelity, dosage and the effects on communication skills.

## Abstract 2

"Through their scoping review, Kajee et al. illustrate how mindfulness training seems to improve distress, depression and anxiety while findings regarding burnout are mixed and too little work has been done regarding empathy."

## 1 INTRODUCTION

There is a growing evidence base showing that healthcare professionals' (HCPs) mental ill health is a cause of diminished professional and personal functioning,^1^, ^2^, ^3^ retention,^4^ quality of patient care^5^, ^6^ and affects the functioning of health care systems.^7^, ^8^, ^9^, ^10^ While this challenge is not new, there is a call to better support the personal and professional health of HCPs.^11^ Mindfulness training (MT) has shown to be of value in supporting mental health and mental wellbeing in the therapeutic setting.^12^ However, our understanding of the applications of MT across HCP groups and career‐stages is less established. The objective of the present study is to provide a scoping review of SRs evaluating the use of MT in improving the mental health, mental wellbeing and communication skills of the different HCPs across their career‐span. This research contributes a novel inquiry in the literature, which is relevant to a HCP education readership by providing established areas of knowledge, while also identifying relevant directions requiring further research.

As the international burden of disease is on a steep upward trajectory, the demands on HCPs are likely to escalate within the coming years.^13^, ^14^ Empirical data suggests that HCPs' mental health can be adversely influenced during both their educational stage, and work.^15^, ^16^, ^17^ In 2019, a report from the British Medical Association (BMA) described ‘a serious mental health crisis among doctors and medical students’ based upon their research in the UK.^18^ In addition, the recent COVID‐19 pandemic has given rise to an increased burden of mental health issues among HCPs, necessitating immediate attention.^19^

While HCP training equips trainees with the requisite knowledge, skills and competencies to treat patients, more could be done to build their self‐awareness skills to ensure they take good care of themselves and to manage highly stressful and burdening occupational situations.^20^, ^21^, ^22^ Healthcare students have been studied extensively in terms of depression and anxiety‐related disorders, as well as their exposure to bullying and academic stress.^21^, ^22^ Suicidal ideation has been reported as high as 6% within the medical student population,^23^ more than double the rate of general population samples.^24^ The burnout syndrome is an established occupational hazard in HCPs,^25^ considered to be due to the long working hours, shift work, large workload, emotional demands, fatigue and trauma associated with work‐related tasks, and a poor management of chronic stress. Issues of mental health, distress and burnout can reduce capacity for empathy and impair communication skills.^26^, ^27^ HCPs' training curricula increasingly includes courses that teach skills and competencies in developing HCPs' capacity to manage their mental health and mental wellbeing, so as to reduce the chances of experiencing mental ill health, stress and burnout.^28^, ^29^

An appropriately trained HCP workforce is essential to functional healthcare systems. HCPs should be trained in personal professionalism,^30^ inter‐professional teamwork,^31^ in addition to patient care.^32^, ^33^ Training in effective communication is necessary, as it is a central component of professionalism.^34^, ^35^, ^36^ Intra‐ and inter‐professional communication is important for delivering effective care to patients, creating a pro‐social clinical environment and supporting professional development.^37^, ^38^, ^39^ The skills of communication, both verbal and non‐verbal, are applicable across the health professions.^39^ All clinical interactions involve some degree of communication.^40^ Communication skills are regarded as a core competency within the healthcare setting^39^, ^40^, ^41^, ^42^ and can be taught as a part of healthcare curriculum.^43^ This training is often done explicitly, and forms part of under‐ and post‐graduate HCP training curricula.^43^, ^44^ Evidence also suggests indirect and experiential teaching of communication skills may occur, for example, through learnt models of behaviour modification.^45^

The general aim of MT is to improve the capacity to remain grounded in the present moment with cultivation of awareness in a non‐judgemental manner.^46^ Mindfulness‐based stress reduction (MBSR) and mindfulness‐based cognitive therapy (MBCT) were developed as secular MT for general populations and public health intereventions.^47^ MT's effects on a wide range of outcomes have been studied, although most frequently on mental health outcomes of psychological distress, anxiety and depression.^29^, ^48^ MT has also been studied in a wide range of professional groups, including those exposed to high levels of chronic stress in the face of cognitively demanding decision‐making, ranging from secondary school teachers^49^ to elite athletes.^50^ This includes within some HCPs from different levels of training.^29^, ^51^, ^52^, ^53^, ^54^ In general, MT is being widely used in healthcare settings with therapeutic purposes, but is not as widely integrated into HCPs' education systems yet.^55^

A recent study indicates that MT may be synergistic with learning aspects of professionalism, such as supportive communication and empathy.^56^ It has been proposed that MT could benefit HCPs by enhancing self‐awareness, improving emotional regulation, promoting active listening, fostering non‐judgmental attitudes and supporting team collaboration.^57^, ^58^, ^59^, ^60^ However, the gaps and limitations of the MT literature across HCP disciplines have not been clearly established yet. Therefore, a further exploration is needed to identify challenges to effectiveness and implementation of MT to enhance the mental health, mental wellbeing and communication skills across HCPs. The broad range of HCP disciplines in the workforce and their potential differences in MT accessibility and usage makes this relevant, especially considering their educational implications.

## 2 METHODS

A scoping review of SRs was chosen as the study design because of the exploratory aim, and the breadth and depth of the literature we expected to uncover. This allowed us to map a large body of literature to address a broad research question, while critically documenting the highest‐level of evidence on the use of MT in HCPs. This process enabled us to build a more general understanding, and also to identify potential gaps. The study was conducted using the methodological framework for scoping reviews developed by Arksey and O'Malley.^61^ The Preferred Reporting Items for Systematic Reviews and Meta‐Analyses (PRISMA)^62^ recommendations were applied in the construction of this scoping review of SRs **(**Appendix S1 **)**. The protocol was pre‐registered in PROSPERO (CRD42020214626) and Open Science Framework.^63^

### 2.1 Search strategy

The search strategy was developed in a comprehensive manner in collaboration with experts in MT, medical and health science education. Terms relating to participants (e.g. HCPs), concepts (e.g. mindfulness), and context (e.g. education), were generated **(**Appendix S2). The complete search strategy is available in Appendix S2. The following 10 databases were included in the search: Ovid MEDLINE, Ovid Embase, Scopus, Cochrane CENTRAL, EBSCOhost CINAHL, Ovid PsycINFO, Web of Science (Core Collection), OpenGrey, TRIP Database and Google Scholar. These databases were chosen based on their relevance to the healthcare sciences, including research in medical and allied health education. Snowballing of reference lists and hand‐searching were also utilised to find additional SRs. Databases were searched from database inception to 22 February 2023. The initial review of titles and abstracts and the full‐text screening were conducted by two independent reviewers (NK and KM). Discrepancies were solved by a third party (JMM).

### 2.2 Inclusion and exclusion criteria

The inclusion and exclusion criteria were developed based on clear scientific rationale (Appendix S3). Studies were considered eligible for inclusion if (1) participants were training and/or qualified HCPs from any country, it (2) evaluates formal standardised, secular MT programs (i.e. MBSR/MBCT/Adapted MBSR/MBCT‐based programs of a minimum of 4 weeks duration^64^), are (3) quantitative and/or qualitative SRs (systematically‐appraised narrative syntheses with or without meta‐analyses), it (4) evaluates outcomes of HCP depression, anxiety, distress, mental wellbeing, burnout, communication skills and empathy, and are (5) English or Spanish language studies. The definitions of key terms are available in Appendix S4. The term ‘HCPs’ was defined broadly, to include a wide variety of disciplines within the healthcare team, that is, all those who participate in the management of patients in the clinical context and contribute to the inter‐professional team or patient management pathway (Table 1).

**TABLE 1:** Healthcare professional categories included.

Medical doctors/medical students
Specialist doctors/training specialist doctors
Nurses/nursing students
Physiotherapist/physiotherapy students
Occupational therapist/occupational therapy students
Speech language hearing therapist/speech language hearing therapy students
Dieticians/dietetics students
Social workers/social work students
Clinical psychologists/psychology students
Counsellors/counselling students
Support workers in healthcare
Allied health worker/allied health student

Studies were excluded if they (1) did not focus solely on standardised, secular MT (<4 weeks intervention), (2) did not evaluate the outcomes of interest in this scoping review (i.e. HCP depression, anxiety, distress, mental wellbeing, burnout, communication skills, empathy), (3) Non‐secular grouped MT and other training ingredients not evaluated in this scoping review (such as non‐secular vipassana meditation, yoga, acceptance and commitment therapy [ACT]), (4) did not meet criteria for a published, peer‐reviewed SR (i.e. integrative, critical and literature reviews excluded), (5) HCPs that are retired/not clinically or career‐engaged at the time of evaluation, or (6) evaluated patient health outcomes or hospital level variables.

### 2.3 Selection, data charting and synthesis of results

Screening was conducted using Covidence software.^65^ Full texts were obtained for all potentially relevant records. The inter‐rater reliability (Cohen's Kappa) for the title and abstract screening was 0.88 and for the full‐text screening was 0.90, reflecting clear inclusion and exclusion criteria that were aligned with the research question.

The primary focus of the review was the effect of MT on the mental health of HCPs. The mental health outcomes were depression, anxiety, distress, burnout and mental wellbeing (Appendix S4 provides theoretical and operational definitions of these concepts). These were selected as they emerged as important themes recurring throughout the literature. The secondary foci of the review were communication skills and empathy (defined in Appendix S4).

Data extraction was conducted using a pre‐piloted data‐extraction table. This table was iteratively refined to capture the information necessary for addressing the research question, thereby facilitating comparison, amalgamation and insight generation through thematic content analysis. A researcher classified the information that was provided in the SRs. A second researcher triangulated these classifications until an agreement was reached^66^—informed by previous literature in the area of MT and implementation science.^67^, ^68^ We assessed a priori for presence of feasibility assessment, acceptability, hospital training climate, length/frequency of home‐based mindfulness practice and teacher training. Study characteristics and outcome findings were also captured, and included the following categories: year of publication, population (including country), number of studies, design, number of participants, MT type, MT duration, outcomes, measures, findings, effect sizes and whether the MT was mandatory within curriculum/voluntary. Finally, we evaluated equity, diversity and inclusion (EDI) parameters to describe how inclusive the MT for HCPs research is (in terms of design and recruitment, data analysis and interpretation, and reporting and dissemination). All these categories enabled us to offer a comprehensive perspective that was not solely focused on program effects. An ‘Evidence‐Gap’ map was generated to summarise the confidence and strength of the evidence as reported by the SRs regarding intervention details (such as ‘fidelity’, as the extent to which the intervention has been delivered in keeping with the original intended programme, also typically referred as ‘adherence’, ‘compliance’, ‘integrity’, ‘faithful replication’, ‘dosage’ and ‘length’^65^, ^66^), as well as the ‘effect durability’, ‘teacher training’, ‘clinical environment’ and EDI considerations.

The AMSTAR2 tool^69^ was utilised for assessment of the methodological quality of included SRs. The SRs were further assessed for risk of bias using the ROBIS tool.^70^ Narrative synthesis of individual and aggregated data from studies is reported. Due to large between‐study heterogeneity of outcome measures and populations, an aggregated statistical analysis of outcome effect sizes was not considered appropriate.^71^

## 3 RESULTS

**FIGURE 1:**
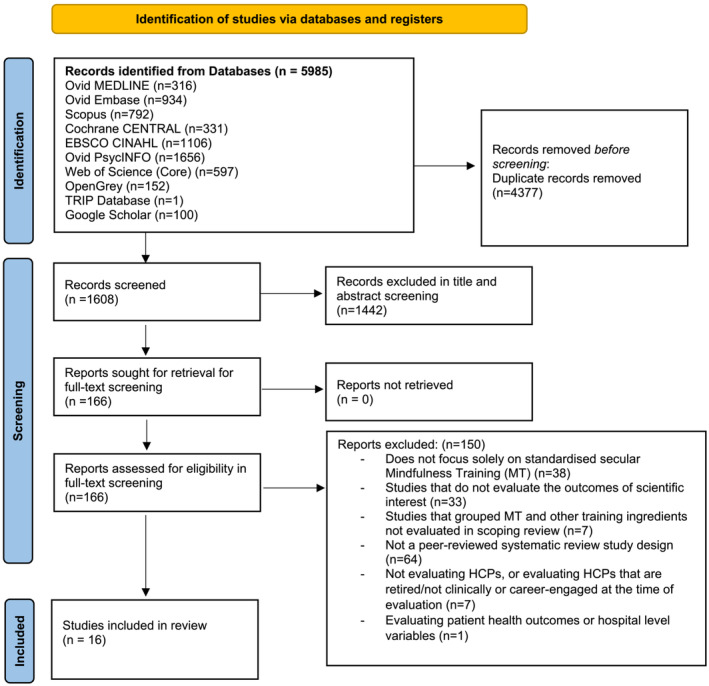
The Preferred Reporting Items for Systematic Reviews and Meta‐Analyses (PRISMA) diagram for the scoping review. [Color figure can be viewed at wileyonlinelibrary.com]

A total of 5985 records were retrieved from the databases on 22 February 2023. Following removal of duplicates (*n* = 4377), the eligible articles (*n* = 1608) were evaluated with title and abstract screening. The title and abstract screening were completed on 26 February 2023, with 166 articles evaluated in subsequent full‐text screening. Full‐text screening was completed on 30 March 2023, with a total of 16 SRs included (Figure 1: PRISMA Diagram for Scoping Review).^59^, ^72^, ^73^, ^74^, ^75^, ^76^, ^77^, ^78^, ^79^, ^80^, ^81^, ^82^, ^83^, ^84^, ^85^, ^86^ A total of *n* = 99 RCTs and *n* = 200 non‐RCTs were included within the SRs analysed. Of the non‐RCTs, *n* = 19 were cross‐sectional studies and *n* = 8 were quasi‐experimental, with the remainder being undifferentiated within SRs. Quantitative SRs (*n* = 15) were the predominant design, with only one qualitative SR (*n* = 1). All studies were published from 2016 onwards.

### 3.1 Participants of included SRs

The SRs collectively evaluate *N* = 21 779 individuals. Potential overlap of studies within SRs was assessed and is accounted for in the calculation. The included SRs (*n* = 16) characteristics are described in Table 2. Participants were predominantly female (±75%). A segment of the studies evaluated HCP students (SR = 7), as either the sole focus of the SR (SR = 4), or in combination with qualified HCPs (SR = 3). These student groups included medical, psychology, nursing and social work students. The qualified HCPs included GPs, medical doctors in speciality training, nurses, psychotherapists, and support or social workers across 12 SRs (*N* = 9308). The study of voluntary programs was higher (SRs = 13) than mandatory training and voluntary training combined (SR = 3). HCPs from lower‐ and middle‐income countries (LMICs) were poorly represented in the data aggregations,^78^, ^84^ with others commenting on the total absence of data from LMICs.^74^, ^80^

**TABLE 2:** Characteristics of SRs included.

Review (year)	Population	No. of studies included (N)	Design	Participants (n=)	MT		Outcomes	Measures	Findings/effect sizes	Curricular component (Y/N)
					Training/CG (#std)	Duration				
Burton et al (2015)^72^	Nur (n = 119) PCP (n = 30) uHCP (n = 135)	9	RCT: 2/9 Other: 7/2	284	MBSR (2/9) MBSR adapted (6/9) Adapted MBSR/MBCT (1/9) C: 2/9 W/L: 2/9 Act: 0 uC: 7/9 Pre/post: 6/9 Quasi: 1/9	10 weeks (1/9) 8 weeks (3/9) 4 weeks (3/9) <4 weeks (2/9)	Burnout	PSS MHPSS DASS SoRLE	Burnout: Medium ES (r = 0.342, CI = 0.202–0.468, p < 0.00002)	N
Cooper et al (2020)^73^	PCS (n = 1863)	22	RCT: 2/22 Other: 20/22 (19/22 cross‐sectional)	1863	MBSR (2/7) MBCT (2/7) Adapted MBSR (1/7) Adapted MBCT (1/7) Adapted MBSR/MBCT (1/7) C: 3/22 W/L: 3/22 uC: 19/22 Pre/Post: 19/22	8 weeks (6/7) 9 months (1/7)	Empathy	IRI (6/22)	Empathy: higher self‐reported levels of mindfulness were associated with less empathic personal distress, r = −0.39, 95% CI (−0.57, −0.18), and greater perspective taking, r = 0.27, 95% CI (0.09, 0.44). An analysis of pre‐post data indicated that MT did not have a significant effect on empathy. Pooled ES for provided for Empathic Concern: hedges g = 0.09, 95% CI: −0.15–0.32; k = 6	N
Daya et al (2018)^74^	MS: 12 studies (n = 1197)	12	RCT: 4/12 Other: 8/12	1197	MBSR: 4/12 Adapted MBCT/MBSR: 1/12 Adapted MBSR: 7/12 C: 5/12 W/L: 4/12 Act: 1/12 uC: 7/12 Pre/Post: 7/12	10 weeks: 1/12 8 weeks: 5/12 7 weeks: 2/12 6 weeks: 2/12 5 weeks: 1/12 4 weeks: 1/12	Burnout depression	DASS (2/12) BDI (4/12) SC‐90R (4/12) MBI (3/12)	Depression: no ES calculations provided. ‘5/9 studies reported significant reductions in depression’. Burnout: 2/3 studies ‘no significant reductions in burnout’	Y
Klein et al (2019)^75^	MD (9 studies) Nur (7 studies) UHCPs (18 studies)	34	RCT: 9/34 Other: 25/34	1439	MBSR (11/34) Adapted MBSR/MBCT (23/34) C: 34/34 W/L: 34/34 uC: 0	8 weeks (13/34) 6 weeks (3/34) 4 weeks (5/34) <4 weeks (13/34)	Burnout (n = 1439)	MBI (25/34) ProQOL (4/34) CBI (3/34) OLBI (2/34)	Burnout: 23/34 studies (67.6%) reported an improvement in measured burnout, however only 4/23 applied a randomised design. No meta‐analysis conducted, no pooled ES (heterogeneous sample, risk of bias high cited)	N
Kriakous et al (2020)^76^	Nur: 7 studies MD: 5 studies SW: 2 studies UHCP: 26	30	RCT: 4/30 Other: 26/30	1053	MBSR: 20/30 Adapted MBSR:10/30* C: 13/30 W/L: 12/30 Act: 1/30 uC: 17/30 Pre/Post: 17/30	Undifferentiated	Burnout	MBI (12/22) * OBI (6/22) CBI (4/22)	Burnout: 13/22 studies significant reduction in burnout 9/22 studies non‐significant reductions in burnout No ES reported	N
Lamothe et al (2015)^77^	PCP (n = 70); n = 68) NS (n = 23) PM/MS (n = 78) UHCP (n = 84) GHCS (n = 31) Nur (n = 41)	7*	RCT: 2/7 Other: 5/7	395	MBSR (2/7) Adapted MBSR (5/7) C: 3/7 W/L: 3/7 uC: 4/7 Pre/Post: 4/7	8 weeks (6/7) 7 weeks (1/7)	Empathy (n = 395)	JSPE (4/7) ECRS (1/7) IRI (2/7)	No meta‐analysis conducted, no pooled ES (heterogeneous outcome measures cited)	N
Liu et al (2023)^78^	Nur (n = 807)	12	RCT: 12 Other: None	807	MBSR (10/12) Adapted MBSR/MBCT (2/12)	4 weeks (5/12) 8 weeks (5/12) 12 weeks (1/12) 13 weeks (1/12)	Anxiety Depression	DASS SDS SAS SCL‐90 HADS HAMA POMS‐TA	Large effect on anxiety. MT significantly higher than control (SMD = 0.91, 95% CL: 0.27–1.55, *p* < 0.05) Furthermore the 8‐weeks intervention category reduced anxiety more significantly (SMD = 1.43, 95% CI: 0.61–2.24) than the 4‐weeks intervention (SMD = 1.03, 95% CI: 0.36–1.71). MT showed large effect on improving depressive symptoms, as compared to control groups (SMD = 1.02, 95% CI: 0.42–1.61, *p* < 0.05). Similarly, to anxiety, depressive symptoms reduced more over the 8‐weeks category (SMD = 1.81, 95% CI: 0.78–2.84) than the 4‐week category of intervention (SMD = 0.82, 95% CI: 0.29–1.35)	Y
Lomas et al (2017)^79^	MD: 9 studies Nur: 16 studies PCS: 24 studies MHP: 8 studies UHCP: DP: 4 studies UHCP: 20 studies	81	RCT: 20/81 Other: 60/21	3805	MBSR (9/81) MBCT (5/81) Adapted MBSR/MBCT (67/81) C: 39/81 W/L: 37/81 Act: 2/81 uC: 42/81 Pre/Post: 42/81 NR: 1/81	8 weeks (30/81) 6 weeks (3/81) Various* (48/81)	Burnout Anxiety Depression	BAI1 (1/18) BAI2 (1/18) DASS (5/18) HADS (3/18) PSWQ (1/18) POMS (2/18) STAI (5/18) CBI (3/28) MBI (19/28) ProQOL (1/28) POMS (3/28) Qualitative interviews/Health Survey (2/28) BDI (1/19) CES‐D (2/19) DASS (6/19) ECQ (2/19) HADS (2/19) POMS (2/19) RRQ (2/19) SC‐90R (2/19)	Burnout: Small to medium ES (Cohen's d = −0.33) Anxiety: Medium ES (Cohen's d = −0.51) Depression: Medium ES (Cohen's d = −0.53) (Confidence Intervals and P‐Values not available)	N
McConville et al. (2017)^59^	MS (n = 769) NS (n = 151) SWS (n = 132) PS (n = 60) PM/MS (n = 78) UHCS (n = 625)	19	RCT: 12/19 Other: 7/19	1815	MBSR (10/19) Adapted MBSR (1/19) Adapted MBSR/MBCT (8/19) C: 19/19 W/L: 17/19 Act: 2/19 uC: 0	8 weeks (10/19) 7 weeks (1/19) 5 weeks (7/19) 4 weeks (1/19)	Anxiety (n = 679) Depression (n = 430) Empathy (n = 138)	JSPE (1/7) ECRS (1/7) IRI (1/7) DASS (1/7) DASS‐21 (3/7) SAS (1/7) BAI 1 (1/7) STAI (5/7) BDI (1/7)	Anxiety: (SMD: −0.44; 95% CI: −0.59 to −0.28; *p* < 0.01) Depression: (SMD: −0.54; 95% CI: −0.83 to −0.26; *p* < 0.01) Empathy: (SMD: 0.39; 95% CI: 0.04 to 0.73; *p* < 0.03)	Y
O′ Driscoll et al (2017)^80^	MS&PS (n = 864) MS (n = 538) PS (n = 74) NS (N = 80)	11	RCT: 9/11 Other: 2/11	1556	Adapted MBSR (9/11) Adapted MBSR/MBCT (1/11) Not detailed (1/11)	4 weeks (1/11) 5 weeks (1/11) 6 weeks (3/11) 7 weeks (1/11) 8 weeks (3/11) 10 weeks (1/11) Not detailed (1/11)	Distress Depression	PSS MBI BDI PANAS DASS‐21	Significant effect on depression scores post‐intervention in two studies (*p* = 0.002 and *p* < 0.006 respectively) Distress was significantly improved (Hedges g = 0.65, 95% CI 0.41, 0.88)	N
Ruiz Fernandes et al (2020)^81^	MD & Nur (n = 232)	9	RCT: 4/9 Other: 5/9	232	Adapted MBSR:2/9 MBSR: 7/9	8 weeks: 7/9 6 weeks: 1/9 10 weeks: 1/9	Distress Stress	PSS	Four studies showed a moderate overall improvement in distress a pooled moderate effect (SMD = 0.65 (0.08–1.22), however statistical heterogeneity was moderate (*I* ^*2*^ = 72%)	N
Scheepers et al (2019)^82^	MD (n = 93) GP (n = 563) USD: (n = 299) IMS (n = 136) EMS (n = 44) R (n = 26) SS (n = 61) P (n = 20) PS (n = 33)	24	RCT: 8/24 Other: 16/24	1275	MBSR (7/24) Adapted; MBSR (6/24) MBCT (2/24) Adapted MBCT (5/24) Adapted MBSR/MBCT (4/24) C: 18/24 W/L: 18/24 Act: 0 uC: 6/24 Pre/Post: 6/24 Quasi: 0/24	10 weeks (1/24) 8 weeks (19/24) 4 weeks (2/24) <4 weeks (2/24)	Burnout Empathy Depression Anxiety	MBI (4/24) OBI (1/24) PSS (2/24) JSPE (4/24) DASS‐21 (3/24) SAS (2/24)	No ES reported. Narrative synthesis Improved depression, anxiety, and empathy. No changes in burnout detected	Y
Suleiman‐Martos et al (2019)^83^	Nur (n = 632) sample size allocations not available	17	RCT 8/17 Other: 9/17	632	MBSR (14/17) * Adapted MBSR (3/17) C: 8/17 W/L: 8/17 uC: 9/17 Quasi: 9/17	12 weeks (1/17) 10 weeks (1/17) 8 weeks (6/17) 6 weeks (4/17) 4 weeks (4/17) 3 weeks (1/17)	Burnout	CBI (1/17) ProQOL (7/17) MBI (9/17)	Burnout: two studies included in the meta‐analysis. Collated data suggests decreased ‘emotional burden’, however too few studies included in the meta‐analysis (sample: n = 90) Emotional Exhaustion: (MD: −1.32; 95% CI: −9.41 to 6.78; *p* < 0.01) Depersonalization: (MD: −1.91; 95% CI: −4.50 to 0.68; *p* < 0.01) Personal Accomplishment: (MD: 2.12; 95% CI: −9.91 to 14.14; p < 0.01)	N
Sulosaari et al (2022)	Nur (n = 1009)	11	RCT: 3/11 Other: 8/11 (Quasi‐experimental)	1009	MBSR (2/11) Adapted MBSR (8/11) Adapted MBCT (1)	5 weeks (1/11) 8 weeks (2/11) 12 weeks (1/11) 5 months (1/11) 6 months (3/11) 9 months (1/11) 13 months (1/11) Not detailed (1/11)	Depression Anxiety Burnout Distress	DASS‐21 PSS PANAS MBI HADS GAD‐7	Reports that 10 of the 11 included studies reported improved depression, anxiety and distress and burnout measures. Reported that 1 study found non‐significant effects on burnout. No pooled effect sizes calculated, nor means and standard deviations of outcome measures	N
Trowbridge et al. (2016)	SW (n = 573)	10	Qualitative studies Phenomenological (1/10) Grounded Theory (3/10) Ethnographic (1/10) Open coding (1/10)	573	Adapted MBSR (2/10) Non‐interventional (8/10)	Not detailed	Empathy	Interviews Surveys Focus Groups Ethnographic fieldwork	Gockel's^87^ “Mid‐level theory” supported within this SR, finding that students taking part in MT: ‘Identified an enhanced ability to stay attentive and emotionally connected with patients’. The description qualitatively of the rich meaning derived by HCP participants, that the development of mindfulness skills contributes to the ‘development of a broad range of therapist characteristics’ and ‘skills of the self that are also applied to others’.	N
Yogeswaran et al. (2021)	MS (n = 99)	2	RCT: None Other: 2/2	99	Adapted MBSR (2/2) Prospective pilot cohort design: 2/2	8 weeks (1/2) 7 weeks (1/2) Delivered online (2/2)	Empathy Distress	PSS JSPE	Significant improvement in distress. Greater improvement in distress outcome at 4‐month F/U. No significant effect on burnout levels. Significant small ES change in empathy	N

*Note*: Effect sizes are reported in accordance with Cohen's criteria.^70^

Abbreviations: Act, Active Control; BAI1, Burns Anxiety Inventory; BAI2, Beck Anxiety Inventory; BDI, Beck Depression Inventory; C, Controlled; CBI, Copenhagen Burnout Inventory; CES‐D, Centre For Epidemiologic Studies – Depression; CG, Control Group; DASS, Depression, Anxiety & Stress Scale; DASS‐21, Depression, Anxiety & Stress Scale Short Version; DP, Disability Professionals; ECQ, Emotional Control Questionnaire; ECRS, Empathy Construct Rating Scale; EMS, Emergency Medicine Specialists; F/U, Follow Up; GHCS, Graduate Health Care Students; GP, General Practitioners; HADS, Hospital Anxiety Depression Scale; IMS, Internal Medical Specialists; IRI, Interpersonal Reactivity Index; JSPE, Jefferson Scale of Physician Empathy; MBI, Maslach Burnout Inventory; MD, Medical Doctors; MHP, Mental Health Professionals; MHPSS, Mental Health Professionals Stress Scale; MS, Medical Students; Nur, Nurses; NR, Not Reported; NS, Nursing Students; OLBI, The Oldenburg Burnout Inventory; P, Psychiatrists; PCP, Primary Care Physicians; PCS, Psychology & Counselling Students; PHQ, Patient Health Questionnaire; PM/MS, Pre‐Medical/Medical students; POMS, Profile of Mood States; ProQOL, Professional Quality of Life Scale; PS, Paediatric Specialists; PSS, Perceived Stress Scale; PSWQ, Penn State Worry Questionnaire; Quasi, Quasi‐Experimental; R, Radiologists; RRQ, Reflection‐Rumination Questionnaire; SAS, Self‐rating Anxiety Scale; SAS*, State Anxiety Scale; SC‐90R, Symptom Checklist 90‐R; SDS, Self‐rating Depression Scale; #SDS, Number of studies; SoRLE, Survey of Recent Life Experiences; SS, Surgical Specialists; STAI, State Trait Anxiety Inventory; SW, Social Workers; SWS, Social Work Students; uC, Uncontrolled; UHCPs, Undifferentiated Health Care Professionals; UHCS, Undifferentiated Health Care Students; USD, Undifferentiated Specialist Doctor.

### 3.2 Type of MT across HCPs

The type of MT investigated was predominantly the 6‐ to 8‐week adapted versions of MBSR and MBCT. Seven SRs evaluated MBSR/MBCT/Adapted versions of both, two SRs evaluated only MBSR, three SRs evaluated MBCT/MBSR and four SRs evaluated MBSR/Adapted MBSR only (Table 2). Four SRs included web, DVD and audio‐visual support as adjuncts within the trainings. Yogeswaran et al.^86^ evaluated the evidence for online MT despite only including two studies in their review, as the area is under‐developed.

### 3.3 Outcomes

#### 3.3.1 Mental health and wellbeing

Seven SRs investigated the relationship between depression and MT.^59^, ^74^, ^76^, ^78^, ^79^, ^80^, ^84^ The SRs reported a small‐to‐medium effect on depression, with 12‐month durability.^74^, ^76^ High‐entry levels of depression in HCPs were noted within the studies; however, pooled values were not provided in SRs.^59^, ^74^, ^79^ Five SRs evaluated anxiety.^59^, ^74^, ^76^, ^79^, ^82^ These studies show a medium‐to‐large effect^88^ of MT in decreasing anxiety symptoms in training and qualified HCPs.

The greatest amount of evidence was within the outcome of distress. Fifty‐three percent of included studies evaluated distress in HCPs receiving MT. The studies show a moderate‐to‐large effect^88^ in the relationship between MT and decreasing distress. This finding was most pronounced in qualified HCPs as compared to their training counterparts.^72^, ^80^, ^84^

Findings of the reviews are equivocal on the effect of MT on burnout in HCPs. Scheepers et al.,^82^ Lomas et al.^79^ and Sulosaari et al.^84^ report a small‐to‐medium effect^88^ of MT on burnout in HCPs. This, however, is contrasted by the findings of Kriakous et al.^76^ and Klein et al.^75^ who find no significant effects in their respective SRs. Scheepers et al.^82^ and Lomas et al.^79^ indicate a positive relationship between MT and wellbeing, with a moderate effect size. The outcome measures are evaluated using a wide range of questionnaires, and there is very little overlap across the studies in the measures applied (Table 2).

#### 3.3.2 Communication skills

Across the evaluated SRs, the effect of MT on communication skills is not adequately examined. Six of the SRs (38% of included SRs) evaluated empathy, which may be considered a para‐communicative skill (Appendix S4). There was a significant increase in empathy within HCPs receiving MT as compared to control groups across four of the included SRs, with two reporting insignificant findings.^59^, ^73^, ^77^, ^79^, ^85^, ^86^ According to Trowbridge et al.,^85^ in the qualitative analyses of the effect of MT on empathic development in social workers, participants reported an ‘enhanced ability to stay attentive and emotionally connected with patients’, and interestingly for the potential meaning‐making in communication skills, they improved ‘skills of the self that are also applied to others’.

Yogaswaran et al.^86^ report this area of inquiry in the realm of online‐interventions targeting empathy in HCPs, as ‘nascent’, and evaluated two studies where the effect on empathy was insignificant. Scheepers et al.,^82^ Lomas et al.^79^ and Lamothe et al.^77^ report an increase in self‐reported empathy following MT; however, no meta‐analyses were conducted. McConville et al.^59^ showed a medium effect of MT on empathy. Cooper et al.^73^ showed that higher self‐reported levels of mindfulness were associated with less empathic personal distress and greater perspective taking, but no significant pre/post changes in empathy were reported.

### 3.4 HCP categories

Across HCP qualified groups, there were comparable effects of MT on mental health and wellbeing. There were subtle differences within empathy, with lower effects in psychotherapy and counselling students receiving MT when compared to medical students.^73^, ^75^ Yogeswaran et al.^86^ note that ‘although it was difficult to maintain engagement with the program with a heavy academic workload, participants were able to recognise the benefits of mindfulness’; however, no studies included in this review evaluated the effect of hospital climate and the barriers to implementing MT. There is a paucity of data evaluating the effect, acceptability and feasibility of MT in other allied HCPs, such as occupational therapists, physiotherapists, dieticians and support workers.

### 3.5 MT in training versus qualified HCPs

The effect of MT does not fundamentally differ for training and qualified HCPs on any of the evaluated outcomes.^74^, ^75^, ^77^ There is evidence to suggest durability within a 12‐month follow‐up in depression, anxiety, empathy and wellbeing across both training and qualified HCPs.^73^, ^74^, ^75^ However, HCPs in training tend to have higher drop‐out rates as compared to qualified HCPs.^73^, ^74^

### 3.6 EDI

The studies were reviewed in terms of demographic characteristics across EDI and accessibility groups in terms of the design and recruitment, data analysis and interpretation, and the reporting/dissemination (Appendix S7 represents the inclusion/exclusion of these characteristics within the SRs). ‘Equity’ and ‘inclusion’ did not feature within any of the included SRs. ‘Diversity’ was discussed by one SR, noting a paucity of diversity in the sample populations included.^85^ ‘Race’ was cited by one SR.^85^ ‘Country’ was described in seven SRs^72^, ^74^, ^75^, ^76^, ^78^, ^82^, ^83^ The distribution of countries, where cited, represented the global populations as follows: North America (50.9%), Europe (20%), Australia (11.8%), Asia (14.5%), South America (2.7%) and Africa (0%). ‘Ethnicity’ was included within three of the SRs.^74^, ^76^, ^86^ Accessibility was included within a single study's discussion regarding CD/DVD/online MT interventions.^86^ Gender was included in 12 studies,^59^, ^74^, ^75^, ^76^, ^77^, ^78^, ^80^, ^81^, ^83^, ^84^, ^86^ but it was only discussed as binaries of ‘female’ or ‘male’, or ‘woman’ or ‘man’, with no non‐binary gender groups nor sexual orientations discussed. ‘Disability’ was included within one study, in a limited capacity.^74^

### 3.7 Methodological quality and risk of bias

Methodological quality of the included SRs on the AMSTAR2 grading^69^ was high, with a range from 6 to 10 (possible range: 0–11), and a median score of 9 (interquartile range = 8,9; Appendices S5 and S6). The overall risk of bias assessed using the ROBIS tool was graded to be low across all the reviews. However, only two reviews evaluated publication bias, and there were concerns of measurement bias of the outcome in three SRs (Appendix S6).^73^, ^74^, ^82^

### 3.8 Evidence gap map

The evidence gaps within the SRs are represented in Table 3. Intervention ‘fidelity’ and ‘teacher training’ were discussed with low confidence and moderate strength of evidence. ‘Dosage’ of the training and ‘effect durability’ were reported with medium confidence and moderate strength of evidence. ‘Length’ of intervention was reported with high confidence and high strength of evidence. ‘EDI’ was reported with low confidence and low strength of evidence. Few studies reported on the attendance rates of participants and practice attrition. The clinical environment of training was unreported across the SRs.

**TABLE 3:** Evidence‐gap map.

	Intervention			Effect durability	Teacher training	Clinical environments	EDI
	Fidelity	Dosage	Length				
Strength of evidence:	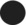	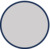	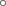	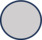	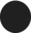	#	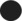
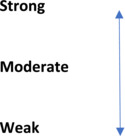							

*Note*: 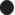 Low confidence 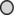 Medium confidence 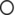 High confidence # *No SR Evidence available*. Amount of data represented by size of circular spheres.

Abbreviation: EDI, equity, diversity and inclusion.

## 4 DISCUSSION

Interest in employing MT to improve mental health, mental wellbeing and communication skills in HCPs is increasing. There is a growing evidence base suggesting that rigorous primary research is needed, for which this study may offer some unique insights.

Most studies suggest MT produces small‐to‐moderate improvements in mental health outcomes, except burnout. Additionally, the evidence suggests that MT produces changes in empathy. Other areas of communication skills, such as attentional control, active listening, recall and conflict resolution (Appendix S4) are not adequately examined. Furthermore, there is an absence of fidelity and quality of MT delivery, teacher training, practice frequency and healthcare climate measurements (Table 2). We suggest that future SRs examine these aspects of MT in HCPs, and also evaluate EDI parameters, which have been neglected so far. In addition, there is a need to expand the originally secular MT research into different spiritual traditions, according to the distinct cultural backgrounds. There is also a paucity of data involving allied HCPs. For instance, occupational health‐, physio‐, speech language hearing therapists, as well as dieticians have not been rigorously studied within a SR. This is of particular interest for educational and clinical advancements as the health sciences become more inter‐professionally dependant within clinical settings.^89^

As the research was predominantly undertaken in self‐selecting groups, there is little data provided from groups who may experience barriers to participation, or for whom participation is compulsory. There is minimal evaluation of sub‐groups who may not benefit from MT, or why there are higher dropout rates in training specific HCPs. A potential hypothesis is that age might be a factor in the interest to explore and cultivate mindfulness skills. Thus, pooled moderation analyses may be particularly valuable in future research.

Several studies did not account for participants with pre‐existing MT, which is important in understanding effects. In addition, few studies documented duration or frequency of home‐based mindfulness practice. This leads to the consideration of the real ‘dose’ of MT received during each intervention. Preceding literature in the MT field suggests that the intervention is impacted by the dose, that is, specific type of practice.^73^ The training and quality of the teachers is largely unexplored. Daya et al.^74^ noted that the presence of medical doctors involved in the teaching process seemed to facilitate uptake of MT and provided understanding of the unique challenges that medical students face when receiving MT. This, however, should be conducted by doctors not directly involved in undergraduate training, as this may present power imbalances and concerns of confidentiality.

Consideration of the location of MT and retreats should also be evaluated, specifically whether it needs to be conducted in a clinical or non‐clinical setting. Location is an important parameter as it could shed light upon why certain HCP groups may have higher drop‐out rates than others (e.g. some locations are more convenient for HCPs working at hospitals). These may be useful for understanding what facilitates/impedes HCPs accessing MT and are essential to develop a translational research model to move the field forward.

Across the healthcare education spectrum, MT is associated with decreased distress, anxiety and depression.^59^, ^78^, ^79^, ^80^, ^82^, ^84^, ^86^ The evidence base for burnout is controversial, with decreased levels reported in some SRs^79^, ^82^, ^84^ and equivocal findings in others.^75^, ^76^ There are concerns in the body of literature that MT may not be sufficient to reverse high burnout, or in HCPs experiencing prolonged burnout, especially in disciplines such as palliative care and oncology.^72^, ^90^ In terms of communication skills, the relationship between burnout and empathy in the context of MT is not analysed. Ultimately, the impact of MT on mental health, mental wellbeing and empathy holds educational significance as long as it may support HCPs in managing stress, preventing burnout, enhancing patient‐centred care, improving communication skills, promoting resilience, and acknowledging the holistic health and wellness of HCPs.

SR quality was collectively strong, and the risk of bias was low on average. At the level of the empirical studies included in SRs, we observed sample sizes tended to be variable. There were few RCTs, and no studies evaluated data duplication. Another concern is the psychometric tools employed to measure the intended outcomes. There is a noticeable lack of consistency and a debatable appropriateness in the use of various measures, such as those related to mental wellbeing and depression. Achieving consistent definitions and operationalisations of outcomes is essential for advancing empirical research in this field.

Within training students, it would be beneficial to investigate a range of professional communicative attributes beyond mental health outcomes. This would help determine the impact of MT on the development of career competencies. As the research base expands, more qualitative studies as well as statistically‐powered RCTs using active control groups will be needed to allow for new syntheses. Of increasing importance will be triangulation of data in these studies, to evaluate the self‐perceived and observed effects of interventions on patient satisfaction, clinical outcomes, communication in the inter‐professional setting and the impact on the clinical culture in the health professions. Durability of mental health outcomes seems promising across the range of a 12‐month follow‐up period. However, further research over longer periods is required. In addition, qualitative research surrounding the lived‐experiences of HCPs practicing MT should be explored in greater detail at the review level.

### 4.1 Strengths and limitations

This is the first scoping review of SRs to explore MT across mental health, mental wellbeing and communication skills outcomes. It examines the full breadth of the health professions and identifies key gaps across the various health care disciplines. MT is not a panacea, and clinical environment ‘cultural’ change remains to be an important aspect of supporting HCPs' personal and professional functioning. Individual MT may be useful for individual self‐care skill development. However, in the case of burnout and work‐place distress, structural issues must be separately addressed. The literature in the area argues for a ‘two‐handed’ approach, in supporting the micro‐, meso‐ and macro‐level challenges^91^ faced by HCPs.^75^, ^76^, ^83^ Future studies evaluating the extent to which individual and group‐delivered mindfulness‐based interventions influence the clinical ‘climate’ are needed. SRs in languages other than English or Spanish were not included in this scoping review, limiting the scope of our search. There is a shortage of qualitative inquiry at this level of review, which limits the research in answering important ecological and lived‐experience questions. The ‘how’, ‘for whom’ and specific circumstances cannot be fully understood by quantitative SRs alone. Some of our study findings, which include the impact of self‐selection within participant groups, the scarcity RCTs, a shortage of high‐powered studies, the limited use of active control groups, predominantly female samples, a lack of data from LMICs, and the extensive absence of EDI parameters and consideration of implementation contexts, impose constraints on our ability to generalise interpretations.

## 5 CONCLUSIONS

MT within the healthcare workforce is growingly researched, and the SRs in the ambit are steadily increasing. Existing studies primarily focus on individual outcomes, without delving deeply into how these individual benefits impact communication within healthcare teams. The evidence base is weighted in support for improving distress, depression, anxiety and mental wellbeing across the HCPs. The effect of MT on empathy seem promising. However, more evidence surrounding burnout and the professional impacts of MT interventions are required, such as the effect on clinical communication skills. Further research should include adequately powered study designs, utilise representative samples from diverse countries and spiritual traditions, and evaluate implementation processes. New research aimed at understanding the qualitative experiences and attitudes of a broad range of students (inclusive of EDI) and qualified HCPs under MT is warranted.

## AUTHOR CONTRIBUTIONS

Nabeela Kajee, Jesus Montero‐Marin, Kate E.A. Saunders, Kearnan Myall, Elinor Harriss and Willem Kuyken conceived the study and collaboratively developed the research protocol. Nabeela Kajee, Jesus Montero‐Marin, Kate E.A. Saunders, Kearnan Myall, Elinor Harriss and Willem Kuyken supported the comprehensive search and extraction of studies. Nabeela Kajee conducted the data synthesis under supervision of Jesus Montero‐Marin, Kate E.A. Saunders and Willem Kuyken. Nabeela Kajee drafted the manuscript. Nabeela Kajee, Jesus Montero‐Marin, Kate E.A. Saunders, Kearnan Myall, Elinor Harriss and Willem Kuyken contributed to interpretation of the data, revised the developing manuscript and approved the final version.

## CONFLICT OF INTEREST STATEMENT

NK and KM are DPhil researchers at the University of Oxford Mindfulness Research Centre (OMC). JMM is associated with the University of Oxford Mindfulness Research Centre. WK is the principal investigator of the University of Oxford Mindfulness Research Centre, holds research grant funding for several projects and receives royalties for several books on mindfulness.

## ETHICS STATEMENT

Not applicable.

## Supporting information


**Appendix S1.** Preferred Reporting Items for Systematic reviews and Meta‐Analyses extension for Scoping Reviews (PRISMA‐ScR) Checklist.
**Appendix S2.** Database Search Strategies (22 February 2023).
**Appendix S3.** Inclusion and Exclusion Criteria of Scoping Review.
**Appendix S4.** Key Definitions of Scoping Review.
**Appendix S5.** Methodological quality (AMSTAR 2) of systematic reviews.
**Appendix S6.** Risk of bias (ROBIS) of systematic reviews.
**Appendix S7.** Table representing the Diversity, Equity, Inclusion (EDI) and demographic components of included population samples.

## Data Availability

Data sharing not applicable to this article as no datasets were generated or analysed during the current study.
