# Supplementary material for: Mindfulness training in healthcare professions: A scoping review of systematic reviews
Source: Med Educ. 2024 Jan 17;58(6):671–86. doi: 10.1111/medu.15293 (PMC11414780; doi:10.1111/medu.15293)
Supplement: Supplementary file 1 — Appendix S1. Preferred Reporting Items for Systematic reviews and Meta‐Analyses extension for Scoping Reviews (PRISMA‐ScR) Checklist. Appendix S2. Database Search Strategies (22 February 2023). Appendix S3. Inclusion and Exclusion Criteria of Scoping Review. Appendix S4. Key Definitions of Scoping Review. Appendix S5. Methodological quality (AMSTAR 2) of systematic reviews. Appendix S6. Risk of bias (ROBIS) of systematic reviews. Appendix S7. Table representing the Diversity, Equity, Inclusion (EDI) and demographic components of included population samples. [file MEDU-58-671-s001.docx]

**Supplemental Digital Appendices**

**Supplemental Digital Appendix 1:** **Preferred Reporting Items for Systematic reviews and Meta-Analyses extension for Scoping Reviews (PRISMA-ScR) Checklist**

| **SECTION** | **ITEM** | **PRISMA-ScR CHECKLIST ITEM** | **REPORTED ON PAGE #** |
| --- | --- | --- | --- |
| **TITLE** | | | |
| Title | 1 | Identify the report as a scoping review. | 1 |
| **ABSTRACT** | | | |
| Structured summary | 2 | Provide a structured summary that includes (as applicable): background, objectives, eligibility criteria, sources of evidence, charting methods, results, and conclusions that relate to the review questions and objectives. | 2 |
| **INTRODUCTION** | | | |
| Rationale | 3 | Describe the rationale for the review in the context of what is already known. Explain why the review questions/objectives lend themselves to a scoping review approach. | 4-5 |
| Objectives | 4 | Provide an explicit statement of the questions and objectives being addressed with reference to their key elements (e.g., population or participants, concepts, and context) or other relevant key elements used to conceptualize the review questions and/or objectives. | 5 |
| **METHODS** | | | |
| Protocol and registration | 5 | Indicate whether a review protocol exists; state if and where it can be accessed (e.g., a Web address); and if available, provide registration information, including the registration number. | 6 |
| Eligibility criteria | 6 | Specify characteristics of the sources of evidence used as eligibility criteria (e.g., years considered, language, and publication status), and provide a rationale. | 6 |
| Information sources* | 7 | Describe all information sources in the search (e.g., databases with dates of coverage and contact with authors to identify additional sources), as well as the date the most recent search was executed. | 6 |
| Search | 8 | Present the full electronic search strategy for at least 1 database, including any limits used, such that it could be repeated. | Supplement |
| Selection of sources of evidence† | 9 | State the process for selecting sources of evidence (i.e., screening and eligibility) included in the scoping review. | Supplement |
| Data charting process‡ | 10 | Describe the methods of charting data from the included sources of evidence (e.g., calibrated forms or forms that have been tested by the team before their use, and whether data charting was done independently or in duplicate) and any processes for obtaining and confirming data from investigators. | 6-8 |
| Data items | 11 | List and define all variables for which data were sought and any assumptions and simplifications made. | Supplement |
| Critical appraisal of individual sources of evidence§ | 12 | If done, provide a rationale for conducting a critical appraisal of included sources of evidence; describe the methods used and how this information was used in any data synthesis (if appropriate). | Supplement |
| Synthesis of results | 13 | Describe the methods of handling and summarizing the data that were charted. | 8-11 |
| **RESULTS** | | | |
| Selection of sources of evidence | 14 | Give numbers of sources of evidence screened, assessed for eligibility, and included in the review, with reasons for exclusions at each stage, ideally using a flow diagram. | Supplement |
| Characteristics of sources of evidence | 15 | For each source of evidence, present characteristics for which data were charted and provide the citations. | 8-11 |
| Critical appraisal within sources of evidence | 16 | If done, present data on critical appraisal of included sources of evidence (see item 12). | 11 |
| Results of individual sources of evidence | 17 | For each included source of evidence, present the relevant data that were charted that relate to the review questions and objectives. | 8-11 |
| Synthesis of results | 18 | Summarize and/or present the charting results as they relate to the review questions and objectives. | 21-29 |
| **DISCUSSION** | | | |
| Summary of evidence | 19 | Summarize the main results (including an overview of concepts, themes, and types of evidence available), link to the review questions and objectives, and consider the relevance to key groups. | 11-14 |
| Limitations | 20 | Discuss the limitations of the scoping review process. | 15 |
| Conclusions | 21 | Provide a general interpretation of the results with respect to the review questions and objectives, as well as potential implications and/or next steps. | 14 |
| **FUNDING** | | | |
| Funding | 22 | Describe sources of funding for the included sources of evidence, as well as sources of funding for the scoping review. Describe the role of the funders of the scoping review. | 15 |

JBI = Joanna Briggs Institute; PRISMA-ScR = Preferred Reporting Items for Systematic reviews and Meta-Analyses extension for Scoping Reviews.

* Where *sources of evidence* (see second footnote) are compiled from, such as bibliographic databases, social media platforms, and Web sites.

† A more inclusive/heterogeneous term used to account for the different types of evidence or data sources (e.g., quantitative and/or qualitative research, expert opinion, and policy documents) that may be eligible in a scoping review as opposed to only studies. This is not to be confused with *information sources* (see first footnote).

‡ The frameworks by Arksey and O’Malley (6) and Levac and colleagues (7) and the JBI guidance (4, 5) refer to the process of data extraction in a scoping review as data charting*.*

§ The process of systematically examining research evidence to assess its validity, results, and relevance before using it to inform a decision. This term is used for items 12 and 19 instead of "risk of bias" (which is more applicable to systematic reviews of interventions) to include and acknowledge the various sources of evidence that may be used in a scoping review (e.g., quantitative and/or qualitative research, expert opinion, and policy document).

*From:* Tricco AC, Lillie E, Zarin W, O'Brien KK, Colquhoun H, Levac D, et al. PRISMA Extension for Scoping Reviews (PRISMAScR): Checklist and Explanation. Ann Intern Med. 2018;169:467–473. [doi: 10.7326/M18-0850](http://annals.org/aim/fullarticle/2700389/prisma-extension-scoping-reviews-prisma-scr-checklist-explanation).

**Supplemental Digital Appendix 2: Database Search Strategies (22 February 2023)**

1. **Database: MEDLINE (Ovid MEDLINE), 1946 to present**

1 mindfulness/

2 meditation/

3 mindful*.ti,ab.

4 "mind ful*".ti,ab.

5 MBSR.ti,ab.

6 MBCT.ti,ab.

7 meditat*.ti,ab.

8 MBI.ti,ab.

9 "mental training".ti,ab.

10 "mental resilience training".ti,ab.

11 1 or 2 or 3 or 4 or 5 or 6 or 7 or 8 or 9 or 10

12 Students, Medical/

13 ((student* or graduate* or undergraduate* or postgraduate*) adj3 (medical or medicine or nursing or physiotherapy* or "occupational therap*" or (speech adj3 language) or dietic* or "social work*" or "allied health")).ti,ab,kw.

14 exp Education, Medical/

15 (medic* adj3 (education or school* or course* or curricul*)).ti,ab,kw.

16 (medic or medics).ti,ab,kw.

17 physician*.ti,ab.

18 doctor*.ti,ab.

19 "medical intern*".ti,ab.

20 "health professional*".ti,ab.

21 (medical adj2 (practitioner* or staff or personnel or profession* or faculty)).ti,ab.

22 clinician*.ti,ab.

23 (clinical adj2 (practitioner* or staff or personnel or profession* or faculty)).ti,ab.

24 ("health care" adj2 (practitioner* or staff or personnel or profession* or faculty)).ti,ab.

25 (healthcare adj2 (practitioner* or staff or personnel or profession* or faculty)).ti,ab.

26 consultant*.ti,ab.

27 (gp or gps or "general practitioner*").ti,ab.

28 "family practitioner*".ti,ab.

29 "house officer*".ti,ab.

30 (andrologist* or anesthesiologist* or anaesthesiologist* or cardiologist* or dermatologist* or diabetologist* or endocrinologist* or epileptologist* or gastroenterologist* or geriatrician* or gerontologist* or hematologist* or haematologist* or hepatologist* or immunologist* or "infectious disease* specialist*" or intensivist*).ti,ab.

31 (internist* or "medical geneticist*" or neonatologist* or nephrologist* or neurologist* or obstetrician* or oncologist* or ophthalmologist* or "orthopedic specialist*" or "orthopaedic specialist*" or "osteopathic physician*" or otolaryngologist* or pathologist* or pediatrician* or paediatrician* or phlebologist* or physiatrist* or psychiatrist* or pulmonologist* or radiologist* or rheumatologist* or surgeon* or urologist* or vaccinologist*).ti,ab.

32 exp Health Personnel/

33 Social Workers/

34 (nurse* or physiotherapist* or "physical therapist*" or "occupational therapist*" or ((speech or language or hearing) adj2 therapist*) or dietician* or "social worker*" or "allied health worker*").ti,ab.

35 12 or 13 or 14 or 15 or 16 or 17 or 18 or 19 or 20 or 21 or 22 or 23 or 24 or 25 or 26 or 27 or 28 or 29 or 30 or 31 or 32 or 33 or 34

36 (((comprehensive* or integrative or systematic*) adj3 (bibliographic* or review* or literature)) or (meta-analy* or metaanaly* or "research synthesis" or ((information or data) adj3 synthesis) or (data adj2 extract*))).ti,ab. or (cinahl or (cochrane adj3 trial*) or embase or medline or psyclit or (psycinfo not "psycinfo database") or pubmed or scopus or "sociological abstracts" or "web of science").ab. or ("cochrane database of systematic reviews" or evidence report technology assessment or evidence report technology assessment summary).jn. or Evidence Report: Technology Assessment*.jn. or ((review adj5 (rationale or evidence)).ti,ab. and review.pt.) or meta-analysis as topic/ or Meta-Analysis.pt.

37 "literature review".ti,ab.

38 36 or 37

39 11 and 35 and 38

1. **Database: Ovid PsycInfo, 1806 to present**

1 mindfulness/ or mindfulness-based interventions/

2 meditation/

3 mindful*.ti,ab.

4 "mind ful*".ti,ab.

5 MBSR.ti,ab.

6 MBCT.ti,ab.

7 meditat*.ti,ab.

8 MBI.ti,ab.

9 "mental training".ti,ab.

10 "mental resilience training".ti,ab.

11 1 or 2 or 3 or 4 or 5 or 6 or 7 or 8 or 9 or 10

12 medical students/

13 exp medical education/ or exp physicians/

14 physician*.ti,ab.

15 doctor*.ti,ab.

16 "medical intern*".ti,ab.

17 "health professional*".ti,ab.

18 (medical adj2 (practitioner* or staff or personnel or profession* or faculty)).ti,ab.

19 clinician*.ti,ab.

20 (clinical adj2 (practitioner* or staff or personnel or profession* or faculty)).ti,ab.

21 ("health care" adj2 (practitioner* or staff or personnel or profession* or faculty)).ti,ab.

22 (healthcare adj2 (practitioner* or staff or personnel or profession* or faculty)).ti,ab.

23 consultant*.ti,ab.

24 (gp or gps or "general practitioner*").ti,ab.

25 "family practitioner*".ti,ab.

26 "house officer*".ti,ab.

27 (andrologist* or anesthesiologist* or anaesthesiologist* or cardiologist* or dermatologist* or diabetologist* or endocrinologist* or epileptologist* or gastroenterologist* or geriatrician* or gerontologist* or hematologist* or haematologist* or hepatologist* or immunologist* or "infectious disease* specialist*" or intensivist*).ti,ab.

28 (internist* or "medical geneticist*" or neonatologist* or nephrologist* or neurologist* or obstetrician* or oncologist* or ophthalmologist* or "orthopedic specialist*" or "orthopaedic specialist*" or "osteopathic physician*" or otolaryngologist* or pathologist* or pediatrician* or paediatrician* or phlebologist* or physiatrist* or psychiatrist* or pulmonologist* or radiologist* or rheumatologist* or surgeon* or urologist* or vaccinologist*).ti,ab.

29 exp Health Personnel/

30 Social Workers/

31 (nurse* or physiotherapist* or "physical therapist*" or "occupational therapist*" or ((speech or language or hearing) adj2 therapist*) or dietician* or "social worker*" or "allied health worker*").ti,ab.

32 12 or 13 or 14 or 15 or 16 or 17 or 18 or 19 or 20 or 21 or 22 or 23 or 24 or 25 or 26 or 27 or 28 or 29 or 30 or 31

33 (((comprehensive* or integrative or systematic*) adj3 (bibliographic* or review* or literature)) or (meta-analy* or metaanaly* or "research synthesis" or ((information or data) adj3 synthesis) or (data adj2 extract*))).ti,ab. or (cinahl or (cochrane adj3 trial*) or embase or medline or psyclit or (psycinfo not "psycinfo database") or pubmed or scopus or "sociological abstracts" or "web of science").ab. or ("cochrane database of systematic reviews" or evidence report technology assessment or evidence report technology assessment summary).jn. or Evidence Report: Technology Assessment*.jn. or ((review adj5 (rationale or evidence)).ti,ab. and review.pt.)

34 meta analysis/

35 exp "literature review"/

36 "literature review".ti,ab.

37 33 or 34 or 35 or 36

38 11 and 32 and 37

1. **Database: Web of Science – Core Collection**

(mindful*  OR  "mind ful*"  OR  mbsr  OR  mbct  OR  meditat*  OR  mbi  OR  "mental training"  OR  "mental resilience training")

AND

((student*  OR  graduate*  OR  undergraduate*  OR  postgraduate* )  near/3  ( medical  OR  medicine  OR  nursing  OR  physiotherapy*  OR  "occupational therap*"  OR  ( speech  near/3  language )  OR  dietic*  OR  "social work*"  OR  "allied health" ))  OR  ( medic*  near/3  ( education  OR  school*  OR  course*  OR  curricul*))  OR ( medic  OR  medics  OR  physician*  OR  doctor*  OR  "medical intern*"  OR  "health professional*"  OR  clinician* )  OR ( medical  near/2  ( practitioner*  OR  staff  OR  personnel  OR  profession*  OR  faculty ))  OR (clinical  near/2  ( practitioner*  OR  staff  OR  personnel  OR  profession*  OR  faculty ))  OR   ("health care"  near/2  (practitioner*  OR  staff  OR  personnel  OR  profession*  OR  faculty ))  OR  ( healthcare  near/2  (practitioner*  OR  staff  OR  personnel  OR  profession*  OR  faculty ))  OR  (consultant*  OR  gp  OR  gps  OR  "general practitioner*"  OR  "house officer*")  OR   (andrologist*  OR  anesthesiologist*  OR  anaesthesiologist*  OR  cardiologist*  OR  dermatologist*  OR  diabetologist*  OR  endocrinologist*  OR  epileptologist*  OR  gastroenterologist*  OR  geriatrician*  OR  gerontologist*  OR  hematologist* )  OR  (haematologist*  OR  hepatologist*  OR  immunologist*  OR  "infectious disease* specialist*"  OR  intensivist* )  OR  ( internist*  OR  "medical geneticist*"  OR  neonatologist*  OR  nephrologist*  OR  neurologist*  OR  obstetrician*  OR  oncologist*  OR  ophthalmologist*  OR  "orthopedic specialist*"  OR  "orthopaedic specialist*"  OR  "osteopathic physician*" )  OR   ( otolaryngologist*  OR  pathologist*  OR  pediatrician*  OR  paediatrician*  OR  phlebologist*  OR  physiatrist*  OR  psychiatrist*  OR  pulmonologist*  OR  radiologist*  OR  rheumatologist*  OR  surgeon*  OR  urologist*  OR  vaccinologist* )  OR   ( nurse*  OR  physiotherapist*  OR  "physical therapist*"  OR  "occupational therapist*"  OR  (( speech  OR  language  OR  hearing )  near/2  therapist*)  OR  dietician*  OR  "social worker*"  OR  "allied health worker*" )

 AND

(( comprehensive*  OR  integrative  OR  systematic* )  near/3  ( bibliographic*  OR  review*  OR  literature ) )  OR   meta-analy*  OR  metaanaly*  OR  "research synthesis"  OR  ( ( information  OR  data )  near/3  synthesis )  OR  ( data  near/2 extract* )  OR   cinahl  OR  ( cochrane  near/3 trial* )  OR  embase  OR  medline  OR  psyclit  OR  ( psycinfo  not  "psycinfo database" )  OR  pubmed scopus  OR  "sociological abstracts"  OR  "web of science"  OR  "cochrane database of systematic reviews"  OR  "evidence report"  OR  "technology assessment"  OR  ( review  near/5  ( rationale  OR  evidence ) )  OR  "literature review"

1. **Database: Ovid Embase, 1974 to present**

1 mindfulness/

2 meditation/

3 mindful*.ti,ab.

4 "mind ful*".ti,ab.

5 MBSR.ti,ab.

6 MBCT.ti,ab.

7 meditat*.ti,ab.

8 MBI.ti,ab.

9 "mental training".ti,ab.

10 "mental resilience training".ti,ab.

11 1 or 2 or 3 or 4 or 5 or 6 or 7 or 8 or 9 or 10

12 Students, Medical/

13 ((student* or graduate* or undergraduate* or postgraduate*) adj3 (medical or medicine or nursing or physiotherapy* or "occupational therap*" or (speech adj3 language) or dietic* or "social work*" or psycholog* or "allied health")).ti,ab,kw.

14 Education, Medical/

15 (medic* adj3 (education or school* or course* or curricul*)).ti,ab,kw.

16 (medic or medics).ti,ab,kw.

17 physician*.ti,ab.

18 doctor*.ti,ab. 210163

19 "medical intern*".ti,ab.

20 "health professional*".ti,ab.

21 (medical adj2 (practitioner* or staff or personnel or profession* or faculty)).ti,ab.

22 clinician*.ti,ab.

23 (clinical adj2 (practitioner* or staff or personnel or profession* or faculty)).ti,ab.

24 ("health care" adj2 (practitioner* or staff or personnel or profession* or faculty)).ti,ab.

25 (healthcare adj2 (practitioner* or staff or personnel or profession* or faculty)).ti,ab.

26 consultant*.ti,ab.

27 (gp or gps or "general practitioner*").ti,ab.

28 "family practitioner*".ti,ab.

29 "house officer*".ti,ab.

30 (andrologist* or anesthesiologist* or anaesthesiologist* or cardiologist* or dermatologist* or diabetologist* or endocrinologist* or epileptologist* or gastroenterologist* or geriatrician* or gerontologist* or hematologist* or haematologist* or hepatologist* or immunologist* or "infectious disease* specialist*" or intensivist*).ti,ab.

31 (internist* or "medical geneticist*" or neonatologist* or nephrologist* or neurologist* or obstetrician* or oncologist* or ophthalmologist* or "orthopedic specialist*" or "orthopaedic specialist*" or "osteopathic physician*" or otolaryngologist* or pathologist* or pediatrician* or paediatrician* or phlebologist* or physiatrist* or psychiatrist* or pulmonologist* or radiologist* or rheumatologist* or surgeon* or urologist* or vaccinologist*).ti,ab.

32 exp Health Personnel/

33 Social Workers/

34 (nurse* or physiotherapist* or "physical therapist*" or "occupational therapist*" or ((speech or language or hearing) adj2 therapist*) or dietician* or "social worker*" or psychologist* or "support worker" or "allied health worker*").ti,ab.

35 12 or 13 or 14 or 15 or 16 or 17 or 18 or 19 or 20 or 21 or 22 or 23 or 24 or 25 or 26 or 27 or 28 or 29 or 30 or 31 or 32 or 33 or 34

36 (((comprehensive* or integrative or systematic*) adj3 (bibliographic* or review* or literature)) or (meta-analy* or metaanaly* or "research synthesis" or ((information or data) adj3 synthesis) or (data adj2 extract*))).ti,ab. or (cinahl or (cochrane adj3 trial*) or embase or medline or psyclit or (psycinfo not "psycinfo database") or pubmed or scopus or "sociological abstracts" or "web of science").ab. or ("cochrane database of systematic reviews" or evidence report technology assessment or evidence report technology assessment summary).jn. or Evidence Report: Technology Assessment*.jn. or ((review adj5 (rationale or evidence)).ti,ab. and review.pt.) or meta-analysis as topic/ or Meta-Analysis.pt.

37 "literature review".ti,ab.

38 36 or 37

39 11 and 35 and 38

1. **Database: EBSCO CINHL**

S1 (MH "Mindfulness")

S2 (MH "Meditation")

S3 TI ( mindful* OR "mind ful*" OR MBSR OR MBCT OR meditat* OR MBI OR "mental training" OR "mental resilience training" ) OR AB ( mindful* OR "mind ful*" OR MBSR OR MBCT OR meditat* OR MBI OR "mental training" OR "mental resilience training" )

S4 S1 OR S2 OR S3

S5 (MH "Students, Medical")

S6 (MH "Education, Medical+")

S7 (MH "Physicians+")

S8 TI ( ((student* or graduate* or undergraduate* or postgraduate*) n3 (medical or medicine)) ) OR AB ( ((student* or graduate* or undergraduate* or postgraduate*) n3 (medical or medicine)) )

S9 TI ( medic* n3 (education or school* or course* or curricul*) ) OR AB ( medic* n3 (education or school* or course* or curricul*) )

S10 TI ( medic or medics or physician* or doctor* or "medical intern*" or "health professional*" or clinician* ) OR AB ( medic or medics or physician* or doctor* or "medical intern*" or "health professional*" or clinician* )

S11 TI ( medical n2 (practitioner* or staff or personnel or profession* or faculty) ) OR AB ( medical n2 (practitioner* or staff or personnel or profession* or faculty) )

S12 TI ( clinical n2 (practitioner* or staff or personnel or profession* or faculty) ) OR AB ( clinical n2 (practitioner* or staff or personnel or profession* or faculty) )

S13 TI ( ("health care" n2 (practitioner* or staff or personnel or profession* or faculty)) ) OR AB ( ("health care" n2 (practitioner* or staff or personnel or profession* or faculty)) )

S14 TI ( (healthcare n2 (practitioner* or staff or personnel or profession* or faculty)) ) OR AB ( (healthcare n2 (practitioner* or staff or personnel or profession* or faculty)) )

S15 TI ( consultant* or gp or gps or "general practitioner*" or "house officer*" ) OR AB ( consultant* or gp or gps or "general practitioner*" or "house officer*" )

S16 TI ( andrologist* or anesthesiologist* or anaesthesiologist* or cardiologist* or dermatologist* or diabetologist* or endocrinologist* or epileptologist* or gastroenterologist* or geriatrician* or gerontologist* or hematologist* or haematologist* or hepatologist* or immunologist* or "infectious disease* specialist*" or intensivist* ) OR AB ( andrologist* or anesthesiologist* or anaesthesiologist* or cardiologist* or dermatologist* or diabetologist* or endocrinologist* or epileptologist* or gastroenterologist* or geriatrician* or gerontologist* or hematologist* or haematologist* or hepatologist* or immunologist* or "infectious disease* specialist*" or intensivist* )

S17 TI ( internist* or "medical geneticist*" or neonatologist* or nephrologist* or neurologist* or obstetrician* or oncologist* or ophthalmologist* or "orthopedic specialist*" or "orthopaedic specialist*" or "osteopathic physician*" or otolaryngologist* or pathologist* or pediatrician* or paediatrician* or phlebologist* or physiatrist* or psychiatrist* or pulmonologist* or radiologist* or rheumatologist* or surgeon* or urologist* or vaccinologist* ) OR AB ( internist* or "medical geneticist*" or neonatologist* or nephrologist* or neurologist* or obstetrician* or oncologist* or ophthalmologist* or "orthopedic specialist*" or "orthopaedic specialist*" or "osteopathic physician*" or otolaryngologist* or pathologist* or pediatrician* or paediatrician* or phlebologist* or physiatrist* or psychiatrist* or pulmonologist* or radiologist* or rheumatologist* or surgeon* or urologist* or vaccinologist* )

S18 S5 OR S6 OR S7 OR S8 OR S9 OR S10 OR S11 OR S12 OR S13 OR S14 OR S15 OR S16 OR S17

S19 S4 AND S18

1. **Database: Scopus**

( TITLE-ABS-KEY ( mindful* OR "mind ful*" OR mbsr OR mbct OR meditat* OR mbi OR "mental training" OR "mental resilience training" ) ) AND ( ( TITLE-ABS-KEY ( ( ( student* OR graduate* OR undergraduate* OR postgraduate* ) W/3 ( medical OR medicine ) ) ) OR TITLE-ABS-KEY ( medic* W/3 ( education OR school* OR course* OR curricul* ) ) OR TITLE-ABS-KEY ( medic OR medics OR physician* OR doctor* OR "medical intern*" OR "health professional*" OR clinician* ) OR TITLE-ABS-KEY ( medical W/2 ( practitioner* OR staff OR personnel OR profession* OR faculty ) ) OR TITLE-ABS-KEY ( clinical W/2 ( practitioner* OR staff OR personnel OR profession* OR faculty ) ) OR TITLE-ABS-KEY ( ( "health care" W/2 ( practitioner* OR staff OR personnel OR profession* OR faculty ) ) ) OR TITLE-ABS-KEY ( ( healthcare W/2 ( practitioner* OR staff OR personnel OR profession* OR faculty ) ) ) OR TITLE-ABS-KEY ( consultant* OR gp OR gps OR "general practitioner*" OR "house officer*" ) OR TITLE-ABS-KEY ( andrologist* OR anesthesiologist* OR anaesthesiologist* OR cardiologist* OR dermatologist* OR diabetologist* OR endocrinologist* OR epileptologist* OR gastroenterologist* OR geriatrician* OR gerontologist* OR hematologist* OR haematologist* OR hepatologist ) OR TITLE-ABS-KEY ( hepatologist* OR immunologist* OR "infectious disease* specialist*" OR intensivist* ) OR TITLE-ABS-KEY ( internist* OR "medical geneticist*" OR neonatologist* OR nephrologist* OR neurologist* OR obstetrician* OR oncologist* OR ophthalmologist* OR "orthopedic specialist*" OR "orthopaedic specialist*" OR "osteopathic physician*" OR otolaryngologist* ) OR TITLE-ABS-KEY ( pathologist* OR pediatrician* OR paediatrician* OR phlebologist* OR physiatrist* OR psychiatrist* OR pulmonologist* OR radiologist* OR rheumatologist* OR surgeon* OR urologist* OR vaccinologist* ) ) ) AND ( LIMIT-TO ( LANGUAGE , "English" ) )

1. **Database: Cochrane (CENTRAL)**

#1 MeSH descriptor: [Mindfulness] explode all trees

#2 MeSH descriptor: [Meditation] explode all trees

#3 mindful* OR "mind ful*" OR MBSR OR MBCT OR meditat* OR MBI OR "mental training" OR "mental resilience training" 7075

#4 #1 or #2 or #3

#5 MeSH descriptor: [Students, Medical] explode all trees

#6 MeSH descriptor: [Education, Medical] explode all trees

#7 MeSH descriptor: [Physicians] explode all trees

#8 ((student* or graduate* or undergraduate* or postgraduate*) near/3 (medical or medicine))

#9 medic* near/3 (education or school* or course* or curricul*)

#10 medic or medics or physician* or doctor* or "medical intern*" or "health professional*" or clinician*

#11 medical near/2 (practitioner* or staff or personnel or profession* or faculty)

#12 clinical near/2 (practitioner* or staff or personnel or profession* or faculty)

#13 ("health care" near/2 (practitioner* or staff or personnel or profession* or faculty))

#14 (healthcare near/2 (practitioner* or staff or personnel or profession* or faculty))

#15 consultant* or gp or gps or "general practitioner*" or "house officer*"

#16 andrologist* or anesthesiologist* or anaesthesiologist* or cardiologist* or dermatologist* or diabetologist* or endocrinologist* or epileptologist* or gastroenterologist* or geriatrician* or gerontologist* or hematologist* or haematologist* or hepatologist* or immunologist* or "infectious disease* specialist*" or intensivist*

#17 internist* or "medical geneticist*" or neonatologist* or nephrologist* or neurologist* or obstetrician* or oncologist* or ophthalmologist* or "orthopedic specialist*" or "orthopaedic specialist*" or "osteopathic physician*" or otolaryngologist* or pathologist* or pediatrician* or paediatrician* or phlebologist* or physiatrist* or psychiatrist* or pulmonologist* or radiologist* or rheumatologist* or surgeon* or urologist* or vaccinologist*

#18 #5 or #6 or #7 or #8 or #9 or #10 or #11 or #12 or #13 or #14 or #15 or #16 or #17

#19 #4 and #18

**Grey Literature**

**8. Database: Trip Database**

(title: medic* or nurs* or therap* or physiotherap* or health* or physician* or doctor* or clinician* or consultant* or gp* or "general practitioner*" or officer* or specialist* or dietician* or social)(title:mindful* or "mind ful*" OR meditat* OR MBSR OR MBCT or mbi OR "mental training" OR "mental resilience training")

**9. Database: Google Scholar**

**Sorted by relevance:**

(mindful*|"mind ful*"|MBSR|MBCT|meditat*|MBI|"mental training"|"mental resilience training")(medic*|nurs*|therap*|physiotherap*|health*|physician*|doctor*|clinician*|consultant*|gp*|"general practitioner*"|officer*|specialist*|dietician*|social)(review*)

**10. Database: OpenGrey**

Screened all results from a simple search for **mindful***

*The search strategies were influenced by the terms used in the following systematic reviews:

**(1) Terms for Mindfulness Based Interventions (MBI):**

*Liu Z, Sun YY, Zhong BL. Mindfulness‐based stress reduction for family carers of people with dementia. Cochrane Database of Systematic Reviews 2018, Issue 8. Art. No.: CD012791. DOI: 10.1002/14651858.CD012791.pub2.* [*https://www.cochranelibrary.com/cdsr/doi/10.1002/14651858.CD012791.pub2/appendices#CD012791-sec2-0016*](https://www.cochranelibrary.com/cdsr/doi/10.1002/14651858.CD012791.pub2/appendices#CD012791-sec2-0016)

**(2) Terms for medical students or practicing medical doctors:**

*Gilligan C, James EL, Snow P, Outram S, Ward BM, Powell M, Lonsdale C, Cushing AM, Silverman J, Regan T, Harvey P, Lynagh MC. Interventions for improving medical students' interpersonal communication in medical consultations. Cochrane Database of Systematic Reviews 2016, Issue 11. Art. No.: CD012418. DOI: 10.1002/14651858.CD012418. https://www.cochranelibrary.com/cdsr/doi/10.1002/14651858.CD012418/appendices#CD012418-sec1-0005*

*Rolfe A, Cash‐Gibson L, Car J, Sheikh A, McKinstry B. Interventions for improving patients' trust in doctors and groups of doctors. Cochrane Database of Systematic Reviews 2014, Issue 3. Art. No.: CD004134. DOI: 10.1002/14651858.CD004134.pub3. https://www.cochranelibrary.com/cdsr/doi/10.1002/14651858.CD004134.pub3/appendices#CD004134-sec1-0010*

**Supplemental Digital Appendix 3: Inclusion and Exclusion Criteria of Scoping Review**

**Inclusion Criteria**

1. Participants are training and/or qualified healthcare professionals (HCPs) from any country
2. Evaluates formal standardised, secular MT programs (i.e. MBSR/MBCT/Adapted MBSR/MBCT-based programs of a minimum of 4-weeks duration of training)
3. Quantitative and qualitative systematic reviews (systematically-appraised narrative syntheses with or without meta-analyses)
4. Evaluates outcomes of HCP depression, anxiety, distress, wellbeing, burnout, communication skills and empathy
5. English-language studies only

**Exclusion Criteria**

1. Does not focus solely on standardised, secular Mindfulness Training (MT) (<4 weeks intervention)
2. Studies that do not evaluate the outcomes of scientific interest in this scoping review (i.e. HCP depression, anxiety, distress, wellbeing, burnout, communication skills, empathy)
3. Studies that grouped MT and other training ingredients not evaluated in this scoping review (such as non-secular vipassana meditation, yoga, acceptance and commitment therapy (ACT))
4. Study not meeting criteria for a published, peer-reviewed systematic review (i.e. integrative, critical and literature reviews excluded)
5. HCPs that are retired/not clinically or career-engaged at the time of evaluation
6. Evaluating patient health outcomes or hospital level variables

**Rationale for inclusion criteria**

| 1. Participants are training and/or qualified healthcare professionals (HCPs) from any country | The systematic review evaluates the HCP population |
| --- | --- |
| 1. Evaluates formal standardised, secular MT programs (i.e. MBSR/MBCT/Adapted MBSR/MBCT-based programs of a minimum of 4-weeks duration of training) | The decision to focus the interventional design on secular, standardised MT is to improve empirical understanding of the effect of these interventions when evaluated at the systematic review level. Interventions which deviated from MT, were excluded to improve accuracy. Non-secular elements were excluded as these do not meet the definition of MT applied to the scoping review conducted |
| 1. Quantitative and qualitative systematic reviews (systematically-appraised narrative syntheses with or without meta-analyses) | The systematic review aimed to appraise and evaluate the effect of MT on specific HCP outcomes |
| 1. Evaluates outcomes of HCP depression, anxiety, distress, wellbeing, burnout, communication skills and empathy | The literature base provides an empirical rationale to better understand the effect of MT on specific mental health outcomes, and the effect on the communicative and empathic skills of HCPs |
| 1. English-language studies only | Due to resource limitations and the language proficiencies of primary authors |

**Rationale for exclusion criteria**

| 1. Does not focus solely on standardised, secular Mindfulness Training (MT) (> 4 weeks intervention) | The decision to focus the interventional design on secular, standardised MT is to improve empirical understanding of the effect of these interventions when evaluated at the systematic review level |
| --- | --- |
| 1. Studies that do not evaluate the outcomes of scientific interest in this scoping review (i.e. HCP depression, anxiety, distress, wellbeing, burnout, communication skills, empathy) | To maintain ‘signal’ in the scoping review, outcomes which did not match the outcomes detailed in the question of the study, were excluded |
| 1. Studies that grouped MT and other training ingredients not evaluated in this scoping review (such as non-secular vipassana meditation, yoga, acceptance and commitment therapy (ACT)) | Interventions which deviated from MT, were excluded to improve accuracy. Non-secular elements were excluded as these do not meet the definition of MT applied to the scoping review conducted |
| 1. Study not meeting criteria for a published, peer-reviewed systematic review (i.e. integrative, critical and literature reviews excluded) | This is a scoping review of systematic reviews. The rationale of the study design is to provide an overview of the highest level of evidence available within this study question. It seeks to synthesize systematically conducted review evidence within the range of a broad area of inquiry. The study provides a novel approach within the HCP MT literature in evaluating these outcomes |
| 1. HCPs that are retired/not clinically or career-engaged at the time of evaluation | The scoping review aims to evaluate the effect of MT on HCPs within the training and career trajectory of the members within the professions |
| 1. Evaluating patient health outcomes or hospital level variables | These outcomes are beyond the scope of the review conducted. This scoping review of systematic reviews evaluates the effect of MT on HCPs |

**Supplemental Digital Appendix 4**: **Key Definitions of Scoping Review**

| **Term** | **Definition** |
| --- | --- |
| Anxiety | Anxiety is defined as disproportionate and significant worry, which may be generalized or situation-specific, and may lead to impairment of function.^1^  The DSM-5^1^ defines generalized anxiety disorder (GAD) as “A) Excessive anxiety and worry (apprehensive expectation), occurring more days than not for at least 6 months, about a number of events or activities (such as work or school performance)  B) The person finds it difficult to control the worry  C) The anxiety and worry are associated with three or more of the following six symptoms (with at least some symptoms present for more days than not for the past 6 months).  Restlessness or feeling keyed up or on edge  Being easily fatigued  Difficulty concentrating or mind going blank  Irritability  Muscle tension  Sleep disturbance (difficulty falling or staying asleep, or restless unsatisfying sleep)  D) The disturbance is not better explained by another mental disorder (e.g., anxiety or worry about having panic attacks in panic disorder, negative evaluation in social anxiety disorder [social phobia], contamination or other obsessions in obsessive-compulsive disorder, separation from attachment figures in separation anxiety disorder, reminders of traumatic events in posttraumatic stress disorder, gaining weight in anorexia nervosa, physical complaints in somatic symptom disorder, perceived appearance flaws in body dysmorphic disorder, having a serious illness in illness anxiety disorder, or the content of delusional beliefs in schizophrenia or delusional disorder).  E) The anxiety, worry, or physical symptoms cause clinically significant distress or impairment in social, occupational, or other important areas of functioning.  F) The disturbance is not attributable to the physiological effects of a substance (e.g., a drug of abuse, a medication) or another medical condition (e.g., hyperthyroidism).”^1^  **Outcome measures within the review:** DASS: Depression, Anxiety & Stress Scale; DASS-21: Depression, Anxiety & Stress Scale Short Version; BAI1: Burns Anxiety Inventory; BAI2: Beck Anxiety Inventory; HADS: Hospital Anxiety Depression Scale; PSWQ: Penn State Worry Questionnaire; STAI: State Trait Anxiety Inventory; SAS: State Anxiety Scale; SAS: Self-rating Anxiety Scale |
| Burnout | According to Maslach’s^2^ “multidimensional theory of burnout”, burnout is connected to the occupational experience, and may be defined by the combination of three specific components: ‘emotional exhaustion’ of the individual; the experience of depersonalization; and a sense of reduction in personal accomplishment at work.  **Outcome measures within the review:** MBI: Maslach Burnout Inventory; CBI: Copenhagen Burnout Inventory; OLBI: The Oldenburg Burnout Inventory; ProQOL: Professional Quality of Life Scale; MHPSS: Mental Health Professionals Stress Scale |
| Communication Skills | Communication skills are defined as the dynamic ability to exchange verbal and non-verbal information proficiently, effectively, and appropriately to other individuals in the professional context.^3^ This scoping review evaluates communication skills broadly, to include personal and interpersonal evaluations of the communication skills of Healthcare Professionals (HCPs) with dimensions of attentional control, active listening, message recall and clinical communicative conflict resolution.  **Outcome measures within the review:** JSPE: Jefferson Scale of Physician Empathy; ECRS: Empathy Construct Rating Scale; IRI: Interpersonal Reactivity Index |
| Depression | Depression^4^, also known as clinical depression or major depressive disorder (MD), is a common and important mood disorder. Those who suffer from depression are known to experience persistent feelings of low mood and hopelessness and show signs of loss of interest in activities previously enjoyed. For a diagnosis of depression to be made, symptoms should be present for a minimum of two weeks duration.  The DSM-5^4^ outlines the following criterion to make a diagnosis of depression: “The individual must be experiencing five or more symptoms during the same 2-week period and at least one of the symptoms should be either (1) depressed mood or (2) loss of interest or pleasure.   1. Depressed mood most of the day, nearly every day. 2. Markedly diminished interest or pleasure in all, or almost all, activities most of the day, nearly every day. 3. Significant weight loss when not dieting or weight gain, or decrease or increase in appetite nearly every day. 4. A slowing down of thought and a reduction of physical movement (observable by others, not merely subjective feelings of restlessness or being slowed down). 5. Fatigue or loss of energy nearly every day. 6. Feelings of worthlessness or excessive or inappropriate guilt nearly every day. 7. Diminished ability to think or concentrate, or indecisiveness, nearly every day. 8. Recurrent thoughts of death, recurrent suicidal ideation without a specific plan, or a suicide attempt or a specific plan for committing suicide.   To receive a diagnosis of depression, these symptoms must cause the individual clinically significant distress or impairment in social, occupational, or other important areas of functioning.”  **Outcome measures within the review:** DASS: Depression, Anxiety & Stress Scale;DASS-21: Depression, Anxiety & Stress Scale Short Version; BDI: Back Depression Inventory; CES-D: Centre For Epidemiologic Studies – Depression; SDS: Self-rating Depression Scale; HADS: Hospital Anxiety Depression Scale |
| Distress | Distress is defined as a cluster of generalised symptoms of psychological stress and unpleasant emotions. Distress is often present in mental illnesses, however may be experienced by individuals not meeting diagnostic criteria for common mental illnesses.^5^  **Outcome measures within the review:** PSS: Perceived Stress Scale; MHPSS: Mental Health Professionals Stress Scale; negative subscales of the Scale of Positive & Negative Experiences and the Positive & Negative Affect Scale. |
| Empathy | Hojat et al.^6^ have defined empathy in the clinical setting as “a cognitive attribute that involves an understanding of the inner experiences and perspectives of the patient, combined  with a capability to communicate this understanding to the patient”  **Outcome measures within the review:** JSPE: Jefferson Scale of Physician Empathy; ECRS: Empathy Construct Rating Scale; IRI: Interpersonal Reactivity Index |
| Mental Health | According to the World Health Organization (WHO), mental health is defined as “a state of wellbeing in which the individual realizes his or her own abilities, can cope with the normal stresses of life, can work productively and fruitfully, and is able to make a contribution to his or her community.” ^7^ |
| Mental Illness | Mental illness is defined as psychiatric conditions which are diagnosed on the background of substantial individual mental symptoms on clinical assessment, associated with loss of function, per the standard diagnostic criteria. This definition accepts the Diagnostic and Statistical Manual of Mental Disorders (DSM-5)^8^ or International Classification of Diseases (ICD) as the gold-standard diagnostic criteria.^9^ |
| Mindfulness Training | Mindfulness is the capacity to intentionally regulate human attention and awareness, non-judgementally and with specific attitudes of mind, in real time, which may be trained using mindfulness training (MT).^10^ This scoping review defines MT as secular, standardised techniques based on MBSR and MBCT. The minimum period accepted for these adaptations is 4-weeks in duration, as these have been shown to be empirically effective in the existing literature base.^11^ |
| Wellbeing | Wellbeing is defined as a multifaceted concept which describes the human experience of flourishing, with minimal impairment in psychological function and an associated satisfaction of life.^12^ The WHO defines the ‘foundations of wellbeing’ in the Geneva Charter for wellbeing as:  “A positive vision of health that integrates physical, mental, spiritual and social well-being” underpinning the importance to move towards a “focus of health promotion on empowerment, inclusivity, equity, and meaningful participation”.^13^  Psychological wellbeing has been described by Ryff et al^14^ as constituted by six facets: personal autonomy, ‘mastery’ within ones environmental, individual growth, positive relationships interpersonally, a sense of purpose in ones’ life, and acceptance of the self.  **Outcome measures within the review:** PHQ: Patient Health Questionnaire; ECQ: Emotional Control Questionnaire |

**References:**

1. Guha M. Diagnostic and statistical manual of mental disorders: DSM-5. Reference

Reviews. 2014

2. Maslach C. A Multidimensional theory of burnout. In: Cooper CL, editor. Theories of Organizational Stress Oxford University Press Inc.; 1999.

3. Allen M, editor. The SAGE encyclopedia of communication research methods. SAGE publications; 2017 Apr 11.

4. Tolentino JC, Schmidt SL. DSM-5 Criteria and Depression Severity: Implications for Clinical Practice. Frontiers in psychiatry. 2018;9:450-.10.3389/fpsyt.2018.00450

5. Cuijpers P, Smits N, Donker T, ten Have M, de Graaf R. Screening for mood and anxiety disorders with the five-item, the three-item, and the two-item mental health inventory. Psychiatry Res. 2009;168:250–5.

6. Hojat M, Gonnella JS, Nasca TJ, et al: The Jefferson scale physician empathy 37 of physician empathy: Further psychometric data and differences by gender and specialty at item level. Acad Med 77(suppl):S58-S60, 2002

7. World Health Organization. Promoting mental health: Concepts, emerging evidence, practice: Summary report. World Health Organization; 2004.

8. Guha M. Diagnostic and statistical manual of mental disorders: DSM-5. Reference Reviews. 2014.

9. Organization WH. International classification of diseases for mortality and morbidity

statistics (11th Revision). 2018.

10. Ludwig DS, Kabat-Zinn J. Mindfulness in medicine. Jama. 2008 Sep 17;300(11):1350-2.

11. Strauss C, Gu J, Montero-Marin J, Whittington A, Chapman C, Kuyken W. Reducing stress and promoting well-being in healthcare workers using mindfulness-based cognitive therapy for life. International Journal of Clinical and Health Psychology. 2021 May 1;21(2):100227.

12. Ryan RM, Deci EL. On happiness and human potentials: a review of research on

hedonic and eudaimonic well-being. Annu Rev Psychol. 2001;52:141-

66.10.1146/annurev.psych.52.1.141

13. World Health Organization. The Geneva charter for well-being. Geneva: WHO; 2021 [cited 2023 April 2]. Available from: [www.who.int/publications/m/item/thegeneva-charter-for-well-being](http://www.who.int/publications/m/item/thegeneva-charter-for-well-being)

14. Ryff CD, Singer B. Psychological Well-Being: Meaning, Measurement, and Implications for Psychotherapy Research. Psychotherapy and Psychosomatics. 1996;65(1):14-23.10.1159/000289026

**Supplemental Digital Appendix 5: Methodological quality (AMSTAR 2) of systematic reviews**

**11 Domains (1 point allocated per domain)**

1. A priori design provided
2. Duplicate study selection and data extraction
3. At least two electronic databases searched
4. Status of publication used as an inclusion criteria
5. List of included and excluded studies provided
6. Characteristics of included studies provided
7. Scientific quality of included studies discussed
8. Scientific quality of the included studies used appropriately to form conclusions
9. Appropriate methods to combine studies
10. Publication biased assessed
11. Conflict of interest included

| **Outcome** | **Author** | **Year** | **D1** | **D2** | **D3** | **D4** | **D5** | **D6** | **D7** | **D8** | **D9** | **D10** | **D11** | **TOTAL** |
| --- | --- | --- | --- | --- | --- | --- | --- | --- | --- | --- | --- | --- | --- | --- |
| Anxiety | Scheepers et al. | 2019 | 1 | 1 | 1 | 1 | 1 | 1 | 1 | 1 | 1 | 0 | 0 | 9 |
|  | Lomas et al. | 2018 | 1 | 1 | 1 | 0 | 0 | 0 | 1 | 1 | 1 | 0 | 0 | 6 |
|  | Kriakous et al. | 2020 | 1 | 1 | 1 | 1 | 1 | 1 | 1 | 1 | 1 | 0 | 0 | 9 |
|  | Daya et al. | 2020 | 1 | 1 | 1 | 1 | 1 | 1 | 1 | 1 | 1 | 0 | 0 | 9 |
|  | Liu et al. | 2023 | 1 | 1 | 1 | 1 | 1 | 1 | 1 | 1 | 1 | 0 | 0 | 9 |
|  | Sulosaari et al. | 2022 | 1 | 1 | 1 | 1 | 1 | 1 | 1 | 1 | 1 | 0 | 0 | 9 |
| Burnout | Scheepers et al. | 2019 | 1 | 1 | 1 | 1 | 1 | 1 | 1 | 1 | 1 | 0 | 0 | 9 |
|  | Lomas et al. | 2018 | 1 | 1 | 1 | 0 | 0 | 0 | 1 | 1 | 1 | 0 | 0 | 6 |
|  | Kriakous et al. | 2020 | 1 | 1 | 1 | 1 | 1 | 1 | 1 | 1 | 1 | 0 | 0 | 9 |
|  | Sulosaari et al. | 2023 | 1 | 1 | 1 | 1 | 1 | 1 | 1 | 1 | 1 | 0 | 0 | 9 |
|  | Klein et al. | 2019 | 1 | 0 | 0 | 1 | 1 | 1 | 1 | 1 | 1 | 1 | 0 | 8 |

| **Outcome** | **Author** | **Year** | **D1** | **D2** | **D3** | **D4** | **D5** | **D6** | **D7** | **D8** | **D9** | **D10** | **D11** | **TOTAL** |
| --- | --- | --- | --- | --- | --- | --- | --- | --- | --- | --- | --- | --- | --- | --- |
| Depression | Lomas et al. | 2018 | 1 | 1 | 1 | 0 | 0 | 0 | 1 | 1 | 1 | 0 | 0 | 6 |
|  | Kriakous et al. | 2020 | 1 | 1 | 1 | 1 | 1 | 1 | 1 | 1 | 1 | 0 | 0 | 9 |
|  | Daya et al. | 2020 | 1 | 1 | 1 | 1 | 1 | 1 | 1 | 1 | 1 | 0 | 0 | 9 |
|  | O’Driscoll et al. | 2017 | 1 | 1 | 1 | 1 | 1 | 1 | 1 | 0 | 1 | 0 | 0 | 8 |
|  | Liu et al. | 2023 | 1 | 1 | 1 | 1 | 1 | 1 | 1 | 1 | 1 | 0 | 0 | 9 |
|  | Sulosaari et al. | 2022 | 1 | 1 | 1 | 1 | 1 | 1 | 1 | 1 | 1 | 0 | 0 | 9 |

| **Outcome** | **Author** | **Year** | **D1** | **D2** | **D3** | **D4** | **D5** | **D6** | **D7** | **D8** | **D9** | **D10** | **D11** | **TOTAL** |
| --- | --- | --- | --- | --- | --- | --- | --- | --- | --- | --- | --- | --- | --- | --- |
| Distress | Scheepers et al. | 2019 | 1 | 1 | 1 | 1 | 1 | 1 | 1 | 1 | 1 | 0 | 0 | 9 |
|  | McConville et al. | 2017 | 1 | 1 | 1 | 1 | 1 | 1 | 1 | 1 | 0 | 1 | 1 | 10 |
|  | Kriakous et al. | 2020 | 1 | 1 | 1 | 1 | 1 | 1 | 1 | 1 | 1 | 0 | 0 | 9 |
|  | Daya et al. | 2020 | 1 | 1 | 1 | 1 | 1 | 1 | 1 | 1 | 1 | 0 | 0 | 9 |
|  | Burton et al. | 2018 | 1 | 1 | 1 | 1 | 0 | 0 | 1 | 1 | 1 | 0 | 1 | 8 |
|  | Yogeswaran et al. | 2021 | 1 | 1 | 1 | 1 | 1 | 1 | 1 | 1 | 0 | 1 | 1 | 10 |
|  | O’Driscoll et al. | 2017 | 1 | 1 | 1 | 1 | 1 | 1 | 1 | 0 | 1 | 0 | 0 | 8 |
|  | Sulosaari et al. | 2022 | 1 | 1 | 1 | 1 | 1 | 1 | 1 | 1 | 1 | 0 | 0 | 9 |
|  | Ruiz Fernandes et al. | 2020 | 1 | 1 | 1 | 1 | 1 | 1 | 1 | 1 | 1 | 0 | 0 | 9 |
| Wellbeing | Kriakous et al. | 2020 | 1 | 1 | 1 | 1 | 1 | 1 | 1 | 1 | 1 | 0 | 0 | 9 |
|  | Daya et al. | 2020 | 1 | 1 | 1 | 1 | 1 | 1 | 1 | 1 | 1 | 0 | 0 | 9 |

| **Outcome** | **Author** | **Year** | **D1** | **D2** | **D3** | **D4** | **D5** | **D6** | **D7** | **D8** | **D9** | **D10** | **D11** | **TOTAL** |
| --- | --- | --- | --- | --- | --- | --- | --- | --- | --- | --- | --- | --- | --- | --- |
| Communication Skills/ Empathy | Lomas et al. | 2018 | 1 | 1 | 1 | 0 | 0 | 0 | 1 | 1 | 1 | 0 | 0 | 6 |
|  | McConville et al. | 2017 | 1 | 1 | 1 | 1 | 1 | 1 | 1 | 1 | 0 | 1 | 1 | 10 |
|  | Cooper et al. | 2020 | 1 | 1 | 1 | 1 | 1 | 0 | 1 | 1 | 1 | 0 | 0 | 8 |
|  | Lamothe et al. | 2015 | 1 | 0 | 0 | 1 | 1 | 1 | 0 | 1 | 1 | 1 | 0 | 7 |
|  | Yogeswaran et al. | 2021 | 1 | 1 | 1 | 1 | 1 | 1 | 1 | 1 | 0 | 1 | 1 | 10 |
|  | Trowbridge et al. | 2016 | 1 | 1 | 1 | 1 | 1 | 1 | 1 | 0 | 1 | 0 | 1 | 9 |

*Key:* 1 = Present. 0 = Absent.

**Supplemental Digital Appendix 6: Risk of bias (ROBIS) of systematic reviews**

Domains:

D1: Bias arising from the randomization process

D2: Bias due to deviation from intended intervention

D3: Bias due to missing outcome data

D4: Bias in measurement of the outcome

D5: Bias in selection of the reported result

Judgement Key:


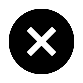
 High concerns


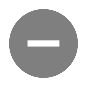
 Some concerns


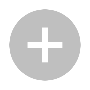
 Low concerns

**
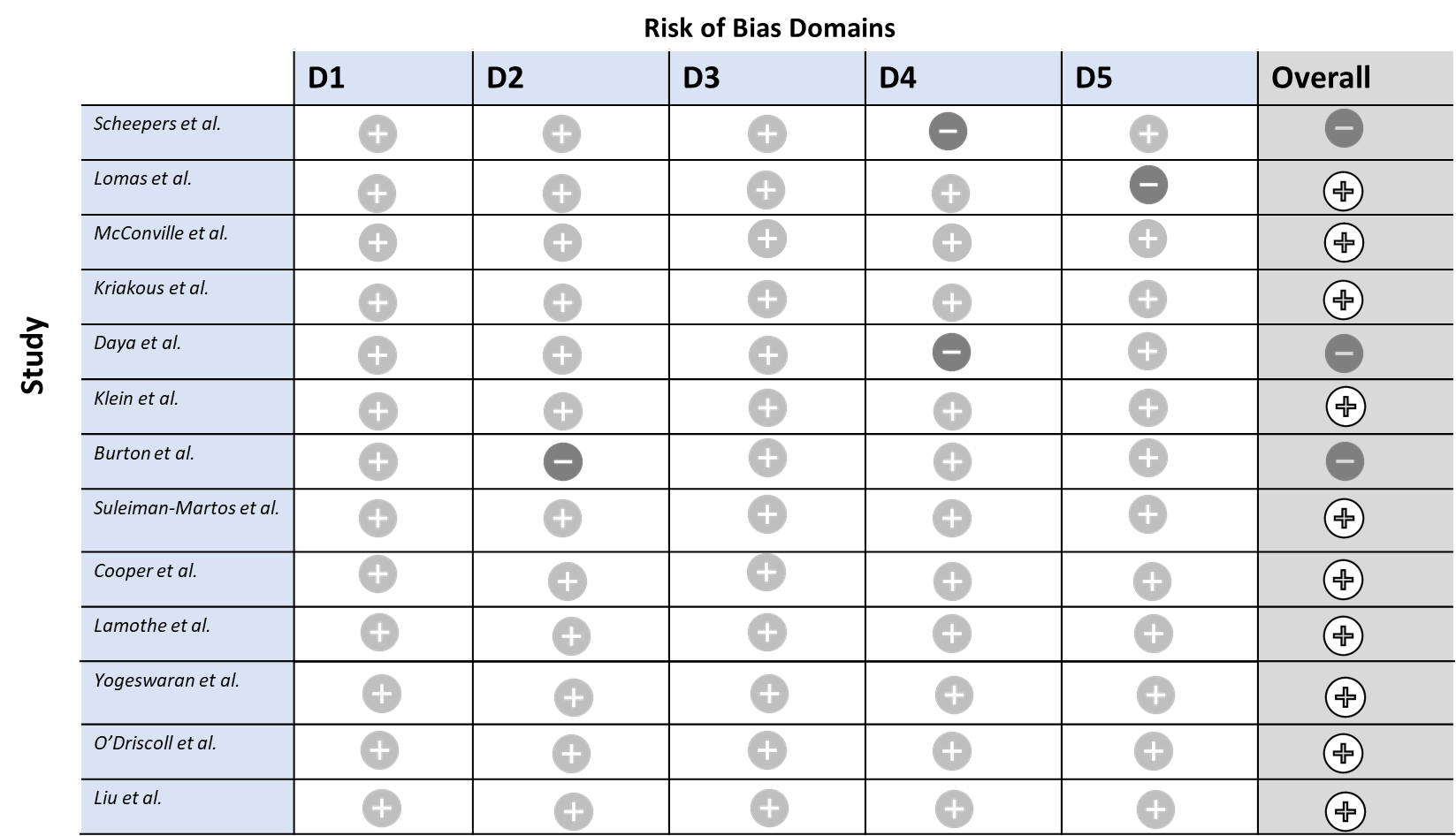
**


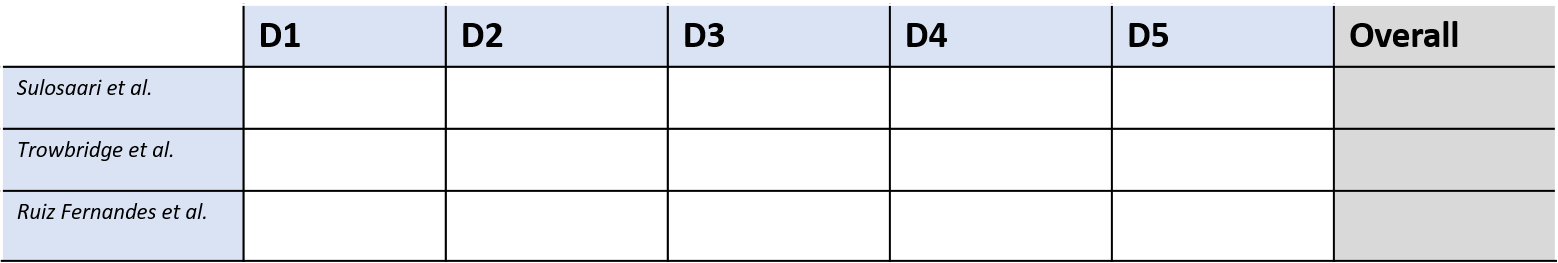

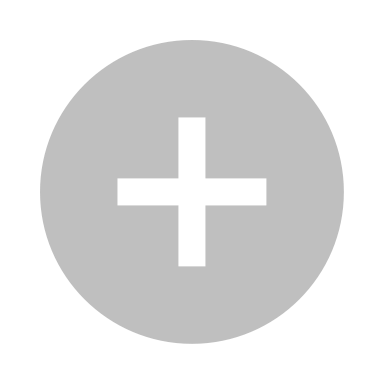

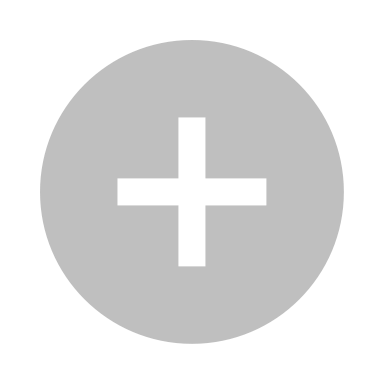

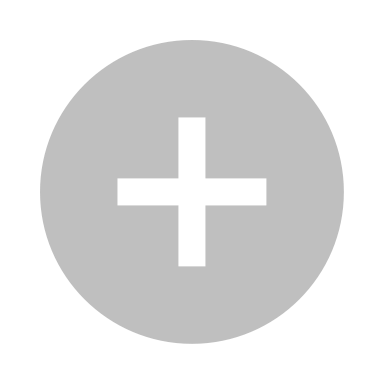

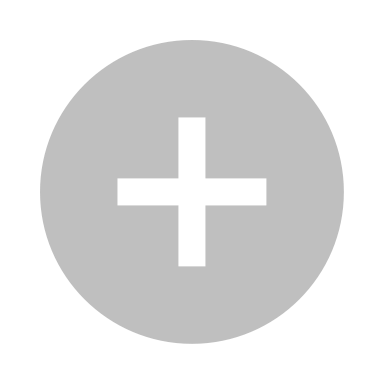

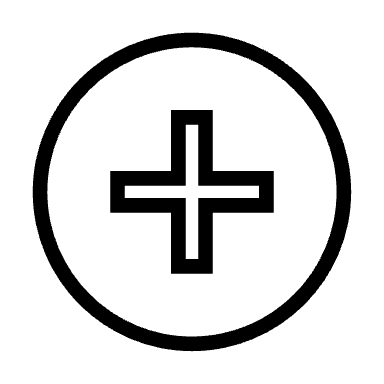

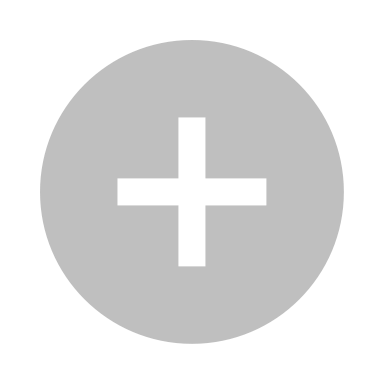

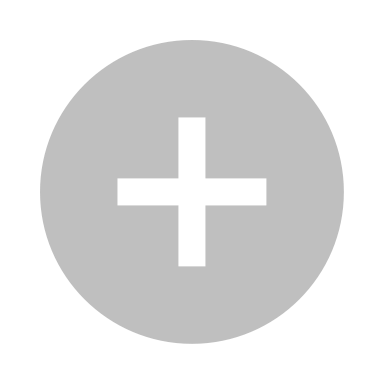

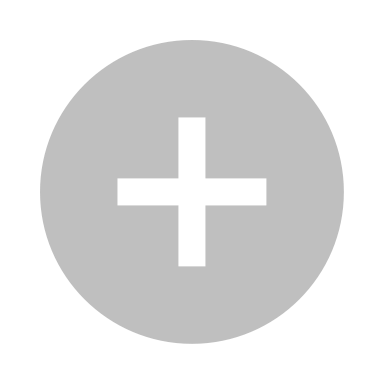

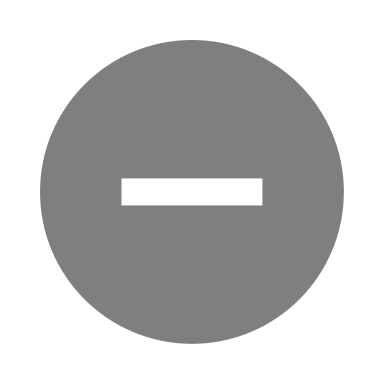

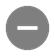

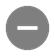

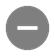

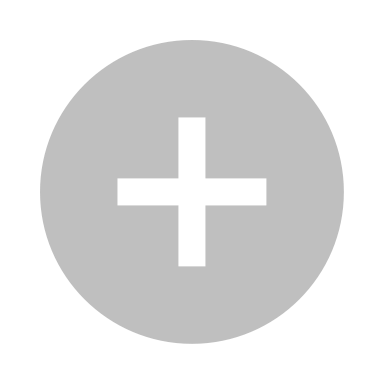

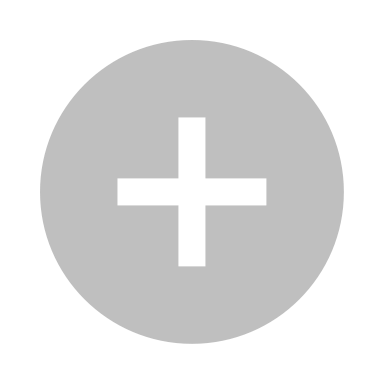

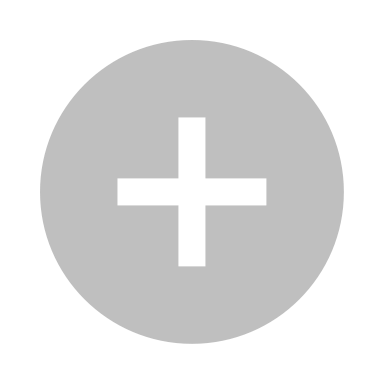

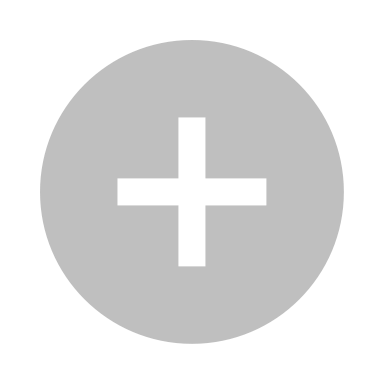

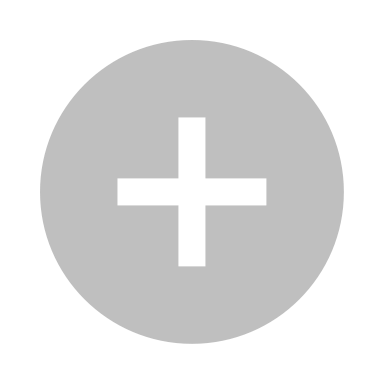

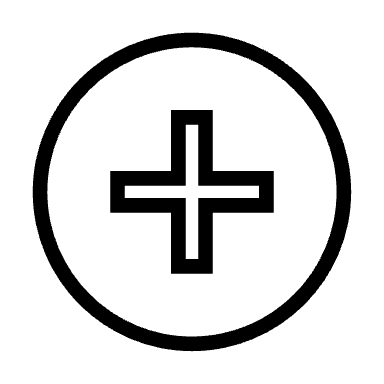


1. Tabulated findings of the critical appraisal of the risk of bias of included systematic reviews.
2. Systematic reviews were not excluded based on the risk assessment.
3. The risk of bias assessment is conducted by two independent researchers (NK and KM) to assess the potential bias of included systematic reviews for the assessment of evidence, disagreements were resolved by a third researcher (JMM). The consensus is presented in tabulated form.

| **Author** | **Equity** | **Diversity** | **Inclusion** | **Race** | **Ethnicity** | **Country** | **Accessibility** | **Gender** | **Disability** |
| --- | --- | --- | --- | --- | --- | --- | --- | --- | --- |
| **Burton et al. (2015)** | None | None | None | None | None | Included | None | Included | None |
| **Cooper et al. (2020)** | None | None | None | None | None | None | None | None | None |
| **Daya et al. (2018)** | None | None | None | Included | Included | Included | None | Included | Included |
| **Klein et al. (2019)** | None | None | None | None | None | Included | None | Included | None |
| **Kriakous et al. (2020)** | None | None | None | None | Included | Included | None | Included | None |
| **Lamothe et al. (2015)** | None | None | None | None | None | None | None | Included | None |
| **Liu et al. (2023)** | None | None | None | None | None | Included | None | Included | None |
| **Lomas et al. (2017)** | None | None | None | None | None | None | None | None | None |
| **McConville et al. (2017)** | None | None | None | None | None | None | None | Included | None |
| **O’Driscoll et al. (2017)** | None | None | None | None | None | None | None | Included | None |
| **Ruiz Fernandes et al. (2020)** | None | None | None | None | None | None | None | Included | None |
| **Scheepers et al. (2019)** | None | None | None | None | None | Included | None | None | None |
| **Suleiman Martos et al. (2019)** | None | None | None | None | None | Included | None | Included | None |
| **Sulosaari et al. (2022)** | None | None | None | None | None | None | None | Included | None |
| **Trowbridge et al. (2016)** | None | Included | None | None | None | None | None | None | None |
| **Yogeswaran et al. (2021)** | None | None | None | None | Included | None | Included | Included | None |
| **Total** | **0** | **1** | **0** | **1** | **3** | **7** | **1** | **12** | **1** |

1. Column terms as defined by the APA EDI Framework: American Psychological Association. (2021b). Equity, diversity, and inclusion framework. https://www.apa.org/about/apa/equitydiversity-Inclusion/equity-division-inclusionframework.pdf

**Supplementary Digital Appendix 7 - *Table representing the Diversity, Equity, Inclusion (EDI) and demographic components of included population samples***
